# Apigenin and Rutaecarpine reduce the burden of cellular senescence in bone marrow stromal stem cells

**DOI:** 10.3389/fendo.2024.1360054

**Published:** 2024-04-04

**Authors:** Dalia Ali, Meshail Okla, Sarah Abuelreich, Radhakrishnan Vishnubalaji, Nicholas Ditzel, Rimi Hamam, Justyna M. Kowal, Ahmed Sayed, Abdullah Aldahmash, Nehad M. Alajez, Moustapha Kassem

**Affiliations:** ^1^ Department of Endocrinology and Metabolism, Molecular Endocrinology & Stem Cell Research Unit (KMEB), Odense University Hospital, University of Southern Denmark, Odense, Denmark; ^2^ Department of Community Health Sciences, College of Applied Medical Sciences, King Saud University, Riyadh, Saudi Arabia; ^3^ Stem Cell Unit, Department of Anatomy, College of Medicine, King Saud University, Riyadh, Saudi Arabia; ^4^ Department of Medical Basic Sciences, College of Medicine, Vision College, Riyadh, Saudi Arabia; ^5^ Institute for Cellular and Molecular Medicine (ICMM), Faculty of Health Sciences, University of Copenhagen, Copenhagen, Denmark

**Keywords:** bone marrow stromal stem cells, osteoblasts, senescence, aging, antioxidants, osteoporosis

## Abstract

**Introduction:**

Osteoporosis is a systemic age-related disease characterized by reduced bone mass and microstructure deterioration, leading to increased risk of bone fragility fractures. Osteoporosis is a worldwide major health care problem and there is a need for preventive approaches.

**Methods and results:**

Apigenin and Rutaecarpine are plant-derived antioxidants identified through functional screen of a natural product library (143 compounds) as enhancers of osteoblastic differentiation of human bone marrow stromal stem cells (hBMSCs). Global gene expression profiling and Western blot analysis revealed activation of several intra-cellular signaling pathways including focal adhesion kinase (FAK) and TGFβ. Pharmacological inhibition of FAK using PF-573228 (5 μM) and TGFβ using SB505124 (1μM), diminished Apigenin- and Rutaecarpine-induced osteoblast differentiation. *In vitro* treatment with Apigenin and Rutaecarpine, of primary hBMSCs obtained from elderly female patients enhanced osteoblast differentiation compared with primary hBMSCs obtained from young female donors. *Ex-vivo* treatment with Apigenin and Rutaecarpine of organotypic embryonic chick-femur culture significantly increased bone volume and cortical thickness compared to control as estimated by μCT-scanning.

**Discussion:**

Our data revealed that Apigenin and Rutaecarpine enhance osteoblastic differentiation, bone formation, and reduce the age-related effects of hBMSCs. Therefore, Apigenin and Rutaecarpine cellular treatment represent a potential strategy for maintaining hBMSCs health during aging and osteoporosis.

## Graphical Abstract

In brief, the natural compounds Apigenin and Rutaecarpine enhanced osteoblastic differentiation of hBMSCs and reduced the burden of cellular senescence and inflammation. Our results suggest a possible role for Apigenin and Rutaecarpine as small molecule antioxidant agents that can be used to prevent impaired osteoblastic functions and bone loss associated with aging and osteoporosis.

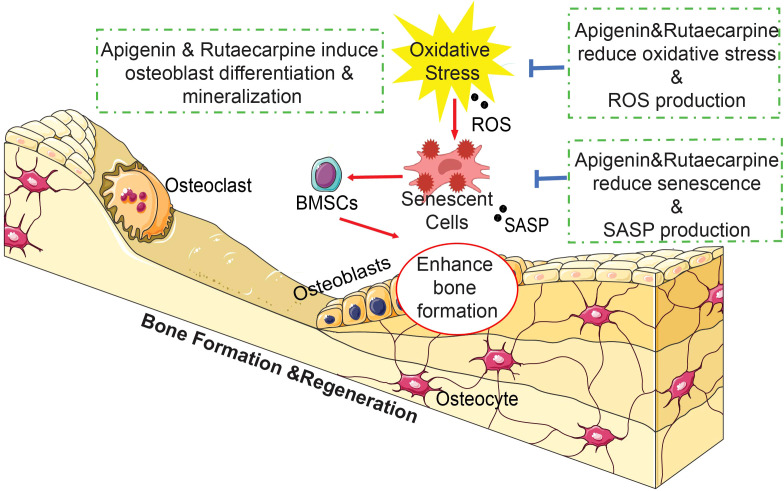

## 1 Introduction

Osteoporosis is a systemic skeletal disease characterized by decreased bone mass and micro-architectural deterioration of bone tissue, leading to bone fragility and increased fracture risk (1). Osteoporosis is caused by imbalance between bone formation and bone resorption during bone remodeling (2). The mechanisms underlying the age-related osteoporosis are either intrinsic cellular mechanisms leading to cellular senescence and affecting osteoblastic functions e.g. telomere shortening, impaired mitochondrial function and increased oxidative stress, or extrinsic factors associated with endocrine aging (3) e.g. menopause or age-related decreased in levels of male sex steroids, GH-IGF system. Both mechanisms affect the cellular and molecular signaling in BMSCs leading to impaired cell proliferation, differentiation, and function (2, 4).

There are currently few drugs that are being used to treat osteoporosis such as Bisphosphonates and Denosumab (5). These medications come with side effects ranging from gastroesophageal irritation to serious problems of increased risk for osteonecrosis of the jaw (6). Preventive strategies include adequate daily intake of calcium, vitamin D supplementation, maintaining optimal body weight, regular physical activity and cessation of smoking and alcohol intake (7). There is an increasing interest in identifying herbal supplements that are affordable and have minimal side effects (8). For example, a Chinese herbal formula (ZD-1) was found to inhibit mineralization and downregulation of several osteogenic markers such as osteocalcin, BMP-2, and osteopontin of hBMSCs (9, 10). Another study reported that Naringin enhanced the osteogenic differentiation via activating the β-catenin pathway (11). Giacomo et al, demonstrated that Tithonia diversifolia inhibited adipogenesis and promoted osteogenesis of hBMSCs via acting as a potent antioxidant (12). Moreover, Resveratrol, a plant derived natural antioxidant and SIRT1 activator, enhances osteogenic differentiation, inhibits adipogenic differentiation, and reduces the senescence-associated phenotype and oxidative stress in aged hBMSCs (13). Apigenin is a major polyphenol in olives and parsley, that was found to inhibit osteoclastogenesis and suppress trabecular bone loss in OVX mice (14). In cultured hBMSCs, Apigenin induced osteogenesis via activating JNK and p38 MAPK signaling pathways (15). Rutaecarpine is derived from the plant *Evodia rutaecarpa* (a dried fruit called ‘Wu-Chu-Yu’ in China) and has been employed in the treatment of cardiovascular diseases (16), obesity (17), gastrointestinal disorders, headache, amenorrhea, and postpartum hemorrhage (18, 19). Additionally, Rutaecarpine inhibits osteoclastogenesis and bone resorption of bone marrow-derived osteoclasts (20).

In the current study, to identify plant-derived natural compounds with significant effects on osteoblast differentiation and bone formation, we conducted a functional osteogenic screening of a natural product library. We identified Apigenin and Rutaecarpine as significant positive regulators of osteoblasts differentiation in hBMSCs and examined their possible underlying molecular mechanisms in the context of aging.

## 2 Materials and methods

### 2.1 Screening natural compounds

Screening Natural compounds library (**Supplementary Table 1**) was purchased from Selleckchem (Selleckchem Inc., Houston, TX, USA, Catalog No. L1400), compounds were dissolved in DMSO at stock concentration of 10mM. Considering previous investigations, which have utilized these compounds within a range of 100nM to 10μM (13, 21, 22), the screening test was conducted with a mid-range concentration of 500nM. For all subsequent experiments, Apigenin and Rutaecarpine were used at a final concentration of 1μM.

### 2.2 Cell culture and osteogenic differentiation

Human Bone Marrow stromal stem cells (hBMSCs) was created by the overexpression of the human telomerase reverse transcriptase gene (hTERT) (23). hBMSCs cell line expresses known markers of primary hBMSCs, exhibits stemness characteristics, and is able to form bone and bone marrow microenvironment when implanted *in vivo* (24). BMSCs were cultured in Minimum Essential Medium (MEM) supplemented with D-glucose 4,500 mg/L, 4 mM l-glutamine, 110 mg/L sodium pyruvate, 10% fetal bovine serum, 1% penicillin–streptomycin (Pen-Strep), and 1% nonessential amino acids. All reagents were purchased from Gibco-Invitrogen (Carlsbad, CA, USA). Cells were incubated in 5.5% CO_2_ incubators at 37^°^C, hBMSCs were cultured to reach 80%–90% confluence before exposing the cells to osteogenic differentiation induction media supplemented with Apigenin or Rutaecarpine at 1μM. Control cells were treated with basal medium containing dimethyl sulfoxide (DMSO) as vehicle. Primary hBMSCs that were used for compounds validation were purchased from Thermo Fisher Scientific. hBMSCs or primary hBMSCs were cultured as noted in the previous section and exposed to osteogenic induction medium (MEM containing 10% FBS, 1% penicillin-streptomycin, 50 mg/ml L-ascorbic acid (Wako-chemicals), 10mM β glycerophosphatase (Sigma-Aldrich), 10 nM calcitriol (1a,25-dihydroxyvitamin D3; Sigma Aldrich), and 100 nM dexamethasone (Sigma-Aldrich) supplemented with the compounds Apigenin or Rutaecarpine at 1μM, media was changed every two days for 10 days. To evaluate the involvement of FAK and TGFβ signaling, hBMSCs were cultured in 96-well plates under osteogenic induction media supplemented with Apigenin, Rutaecarpine or vehicle control and were additionally supplemented with FAK inhibitor (PF-573228, at 5 μM) (Sigma-Aldrich) or transforming growth factor β (TGFβ) signaling inhibitor (SB505124, at 1 μM) (Sigma-Aldrich), media was replaced every 2 days. ALP quantification for osteogenesis was performed on day10.

### 2.3 Primary hBMSCs

Bone marrow samples were collected from female femur of two healthy young donors (Age 25 and 26 years old) and two aged osteoporotic patients (Age 91 and 86 years old) undergoing routine orthopaedic surgeries at the Department of Orthopaedic and Traumatology, Odense University Hospital. The subjects received oral and written project information and signed written consent. The study was approved by the Scientific Ethics Committee of Southern Denmark (project ID: S-20160084). Our selection of the aged patients for this study was based on their BMSCs’ low osteogenic differentiation capacity, as identified in previously published studies (25, 26), as we aim to explore the efficacy of Apigenin and Rutaecarpine in enhancing osteogenic differentiation capacity. Primary hBMSCs were obtained from mononuclear cell population isolated from bone marrow samples following gradient centrifugation in lymphoprep, through plastic adherence. The cells were cultured in MEM media supplemented with 10% foetal bovine serum (FBS) and 1% penicillin/streptomycin (P/S). When the first adherent cells were observed, the media was changed to MEM media supplemented with 10% FBS, 1% P/S, 1% GlutaMAX, 1% sodium pyruvate and 1% non-essential amino acids (S-MEM growing medium). These primary hBMSCs were cultured in 37°C in humidified 5% CO_2_ incubator. Cells from each participant were cultured separately, and only cells from passage two were utilized in the experiments conducted in this study. In primary culture experiments, “n=2” corresponds to the number of subjects per experimental group. In ALP test, each subject contributed 6 technical replicates, resulting in a total of 12 observations. In qPCR, each subject contributed 4 technical replicates, resulting in a total of 8 observations.

### 2.4 Evaluation of osteoblast differentiation

To quantify alkaline phosphatase (ALP) activity in control and osteoblast-differentiated hBMSCs the BioVision ALP activity colorimetric assay kit (BioVision, Inc., Milpitas, CA, http://www.biovision.com/) was used with some modifications. Cells were cultured in 96-well plates under normal or osteogenic induction conditions supplemented with Apigenin or Rutaecarpine at 1μM. On day 10, wells were rinsed once with PBS and were fixed using 3.7% formaldehyde in 90% ethanol for 30 seconds at room temperature. Then the fixative reagent was removed and 50 μl of p-nitrophenyl phosphate solution was added to each well and incubated for 20–30 minutes in the dark at room temperature until a clear yellow color is developed. Reaction was subsequently stopped by adding 20 μl of stop solution. Optical density was then measured at 405nm using a SpectraMax/M5 fluorescence spectrophotometer plate reader. Cell viability was measured using alamarBlue assay according to the manufacturer’s recommendations (Thermo Fisher Scientific). Cell viability was taken in consideration when performing ALP quantification activity on osteogenic differentiated cells. In brief, AlamarBlue was added at ratio of 10% from the volume of the media added on cultured cells in 96-well plates of osteogenic differentiated cells. Plates were incubated in the dark at 37°C for 1h. Reading was subsequently taken using fluorescent mode (Ex 530 nm/Em 590 nm) using BioTek Synergy II microplate reader (BioTek Inc., Winooski, VT, USA).

For ALP staining, cells were washed in PBS, fixed in acetone/citrate buffer and incubated with ALP substrate solution (naphthol AS-TR phosphate 0.1M Tris buffer, pH 9.0) for 1 h at room temperature and subsequently images were taken using an EVOS Cell Imaging System (Thermo Fisher Scientific).

Alzarin Red S staining (ALZR) (ScienCell Research Laboratories, Cat No 0223, San Diego, CA, USA) was used to stain for calcium deposits, which are indicators of mature osteocytes, on day 14 of osteogenic differentiation and upon exposure to Apigenin or Rutaecarpine and according to manufacturer’s protocol. Cells were washed twice with PBS then were fixed with 4% Paraformaldehyde in PBS for 15 min at room temperature, then washed three times with distilled water then added 1ml of 2% ALZR stain to each well for 30 mints then final wash with distilled water at least 3 times before taking images. Images were captured using an EVOS Cell Imaging System (Thermo Fisher Scientific).

### 2.5 Quantitative real-time qPCR

Total RNA was isolated from cell pellets after 10 days of osteogenic differentiation using the Total RNA Purification Kit (Norgen Biotek Corp., Thorold, ON, Canada, https://norgenbiotek.com/) according to the manufacturer’s protocol. The concentrations of total RNA were measured using NanoDrop 2000 (Thermo Fisher Scientific). cDNA was synthesized using 500 ng of total RNA. The Thermo Fisher Scientific High-Capacity cDNA Transcription Kit was used according to manufacturer’s protocol. Expression levels of osteoblast-related genes (**Supplementary Table 2**) were quantified using the ViiA 7 Real-Time PCR device (Thermo Fisher Scientific). Expression was quantified using Fast SYBR Green Master Mix and a ViiA 7 Real-Time PCR device (Thermo Fisher Scientific). The 2DCT value method was used to calculate relative expression, and analysis was performed as previously described (27).

### 2.6 DNA microarray gene expression profiling

A total of 150 ng RNA was labelled using low input Quick Amp Labeling Kit (Agilent Technologies, Santa Carla, CA, USA) and then hybridized to the Agilent Human SurePrint G3 Human GE 8x60k microarray chip (Agilent Technologies, Santa Carla, CA, USA). All microarray experiments were performed at the Microarray Core Facility (Stem Cell Unit, King Saud University College of Medicine, Riyadh, Saudi Arabia). The extracted data were normalized and analyzed using GeneSpring 13.0 software (Agilent Technologies, Santa Carla, CA, USA). Pathway analysis was performed using the Single Experiment Pathway analysis feature in GeneSpring 13.0 (Agilent Technologies Agilent Technologies, Santa Carla, CA, USA). Two-fold cut-off and a p< 0.05 were used to enrich for significantly changed transcripts.

### 2.7 Western blot analysis

hBMSCs were seeded until reaching 60-80% confluence before incubation in serum reduced medium (0.2% FBS), low glucose MEM medium for 6 hours prior to treatment with 1μM of Apigenin, Rutaecarpine or DMSO-vehicle control in osteogenic induction media. Protein samples were harvested at 0, 30, 60, 120 & 240 minutes after treatment. Briefly, cells were washed in PBS and were lysed in RIPA buffer (Invitrogen) supplemented with protease inhibitors (Roche). After 30 min incubation at 4°C, samples were centrifuged for 10 min at 12,000 rpm, 4°C. Protein concentration was determined using Pierce Coomassie Plus Bradford assay (Thermo Fisher Scientific), and equal amounts of proteins were loaded on a 10% polyacrylamide gel (Invitrogen). Blotted nitrocellulose membranes were incubated overnight with antibodies against p-FAK, FAK, p-ERK, ERK2, p-SMAD2, SMAD2 & Actin (Cell Signaling) at 4°C. Membranes were incubated with HRP conjugated anti-mouse or anti-rabbit secondary antibody (Santa Cruz Biotechnology) for 45 min at room temperature, and protein bands were visualized with Amersham ECL chemiluminescence detection system (GE Healthcare Bio-Sciences Corp).

### 2.8 Senescence-associated β-galactosidase (β-gal) staining

To investigate the possible protective role of Apigenin or Rutaecarpine treatmement on senescent cells, we used a commercial kit for β-gal staining (Cell Signaling TechnologyNetherlands, Cat# 9860). BMSCs were cultured in black 96-well clear bottom plate and were treated with Apigenin or Rutaecarpine at 1μM for 2 days. After that cells were differentiated using osteogenic induction media for one week with vehicle control DMSO or Apigenin or Rutaecarpine with or without 50μM TBHP. Cells were washed with PBS, and then were fixed for 10 minutes at room temperature. After fixation the cells were rinsed with PBS and incubated with β-gal staining solution (pH = 6.0) at 37^°^C in dry incubator (none-CO_2_) overnight. The blue color as a reaction result of senescence was monitored after 10-12h. Images of cells were captured with inverted microscope under bright field.

### 2.9 Cellular reactive oxygen species (ROS) detection

A commercial kit of DCFDA (2,7-dichloro-dihydro-fluorescein diacetate; Abcam, Cambridge, MA) was used to measure the intracellular ROS level. hBMSCs were seeded at 2.5x10^4^ cells/well into a black 96 well plate with a clear bottom and were allowed to adhere. First, cells were treated with Apigenin or Rutaecarpine at 1μM for 2 days and then exposed to tert-butyl hydrogen peroxide (TBHP) at 55µM for 2 hours. Next, cells were loaded with DCFDA according to the manufacturer’s protocol and incubated for 45 minutes at 37 °C. After that, DCFDA was removed, and experimental conditions were added again to the cells for 15 minutes. Then, the fluorescent intensity was measured at Excitation 485nm and Emission 535 nm using SpectraMax M5 (Molecular Devices).

### 2.10 Chick femur and micro-computed tomography scanning (μCT)

Ex vivo cultures of embryonic, day 11 (E11) and 13 (E13), chick femurs were performed as described previously (28). In brief, control non-induced femurs were cultured in the basal culture media with ascorbic acid 2-phosphate (100 mM), while other femurs were cultured in osteogenic induction medium along with either DMSO or Apigenin or Rutaecarpine at 1μM. All the femurs placed onto Millicell inserts (0.4-mm pore size, 30-mm diameter; Millipore) in six-well tissue culture plates containing 1mL media per well at the liquid/gas interface. Femurs were cultured for 10 days at 37°C, 5% CO_2_, with media changed every 24 h. Femurs were then fixed in 4% paraformaldehyde (PFA) for 24hr. The chick femurs were micro-CT imaged in air using a vivaCT40-scanner (SCANCO Medical AG, Brüttisellen, Switzerland). Samples were scanned with 70kV, 114µA, and a sampling time of 300ms. Three-dimensional images were reconstructed and analyzed at a resolution of 10.5 µm isotropic voxels using the software supplied by the manufacturer of the scanner.

### 2.11 Statistical analysis

Statistical analyses were performed on Prism 9 (GraphPad Software). Bar graphs are shown as mean % ± *SEM*, and statistical significance between groups was determined by at least 2 independent experiments. The statistical significance was determined by unpaired t-test and one-way ANOVA. All results are compared to DMSO-control unless otherwise stated by the line arrow. *P* value < 0.05 was considered statistically significant.

## 3 Results

### 3.1 Effect of Natural Compounds on Osteogenic Differentiation of hBMSCs

**Figure 1:**
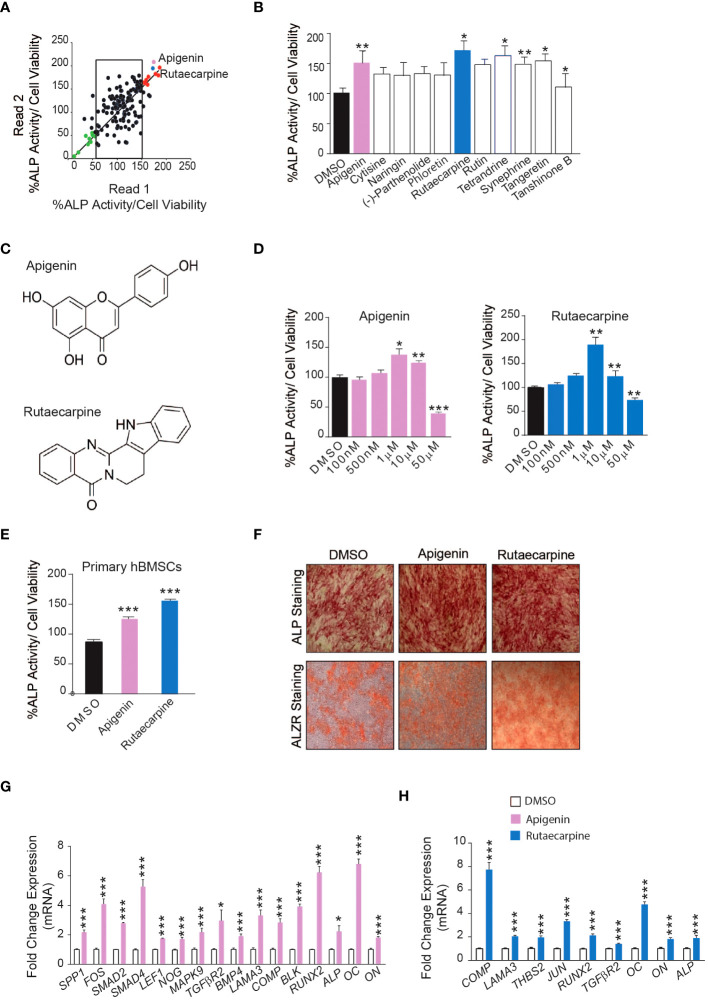
Screening of 143 natural compounds library on osteoblast differentiation revealed significant impact of Apigenin and Rutaecarpine on osteogenesis **(A)** Quantification of Alkaline phosphatase (ALP) activity in 144 compounds treated hBMSCs vs control-treated cells at a dose of 500nM. **(B)** Quantification of Alkaline phosphatase (ALP) activity in 11 compounds at 500nM **(C)** Chemical structure of Apigenin and Rutaecarpine **(D)** Dose response effect of Apigenin, Rutaecarpine on osteogenic differentiation of hBMSCs via quantification of ALP activity at concentrations of 100nM, 500nM, 1μM, 10μM & 50μM, (n=6) from two independent experiments **(E)** Quantification of Alkaline phosphatase (ALP) activity in primary hBMSCs treated with Apigenin or Rutaecarpine or Vehicle control cells at 1μM, (n=12) from two independent experiments **(F)** ALP staining, upper panel (4x magnification) and alizarin red staining for mineralized matrix formation, lower-panel (4x magnification). **(G)** qRT-PCR of a panel of osteoblast-related genes in the presence of Apigenin compared to vehicle control, normalized to β-actin. Data are presented as mean fold changes ± SEM compared with vehicle-treated controls; n = 6 from two independent experiments **(H)** qRT-PCR of a panel of osteoblast-related genes in the presence of Rutaecarpine compared to vehicle control, normalized to β-actin. Data are presented as mean fold changes ± SEM compared with vehicle-treated controls; n = 6 from two independent experiments. Data are presented as mean ± SEM, from two independent experiments, using two-tailed unpaired Student’s t test. (*P< 0.05, **P< 0.005, ***P< 0.0005). All results are compared to DMSO-control unless otherwise stated by the line arrow. Data without the line arrow indicates no statistical significance.

Initially, a library of 143 natural compounds (**Supplementary Table 1**) were screened for thier effect on osteoblastic differentiation of hBMSCs at a dose of 500nM. Cells were continuously exposed to compounds during osteoblastic differentiation media and were assessed by (ALP activity) (**Figure 1A**). Eleven of the most potent significant compounds were chosen and assessed their effects on ALP activity as shown in (**Figure 1B**). Two compounds were chosen, Apigenin and Rutaecarpine as they exerted pronounced effects (**Figure 1C**) and conducted a dose response effect of the compounds on osteoblast differentiation as estimated by ALP activity (**Figure 1D**) that revealed 1μM is the optimum dose to induce osteoblast differentiation of cultured hBMSCs. Apigenin and Rutaecarpine were chosen to be further investigated and at dose of 1μM, results were validated in primary hBMSCs (**Figure 1E**). Similarly, the intensity of ALP staining was higher in Apigenin and Rutaecarpine-treated hBMSCs compared to vehicle-treated control cells (Figure1F, upper panel). In addition, *in vitro* mineralization as evidenced by ALZR staining, was more intense in Apigenin and Rutaecarpine-treated hBMSCs compared to vehicle-treated control cells (**Figure 1F**, lower panel). To identify molecular mechanism mediating enhanced osteoblast differentiation in Apigenin and Rutaecarpine treated cells, we evaluated mRNA levels of selected osteoblastic genes panel using mRNA from hBMSCs post osteogenic differentiation with Apigenin (**Figure 1G**) and Rutaecarpine (**Figure 1H**). Employing osteogenesis-related Apigenin treatment revealed significant up-regulation in: (SPP1, FOS, SMAD2, SMAD4, LEF1, NOG, MAPK9, TGFβR2, BMP4, LAMA3, COMP, BLK, RUNX2, ALP, OC and ON). Rutaecarpine treatment significantly revealed up regulation of osteoblastic markers of: COMP, LAMA3, THBS2, JUN, RUNX2, TGFβR2, OC, ON and ALP.

### 3.2 Genes and pathways differentially regulated in osteoblasts by Apigenin and Rutaecarpine treatments

#### 3.2.1 Apigenin

**Figure 2:**
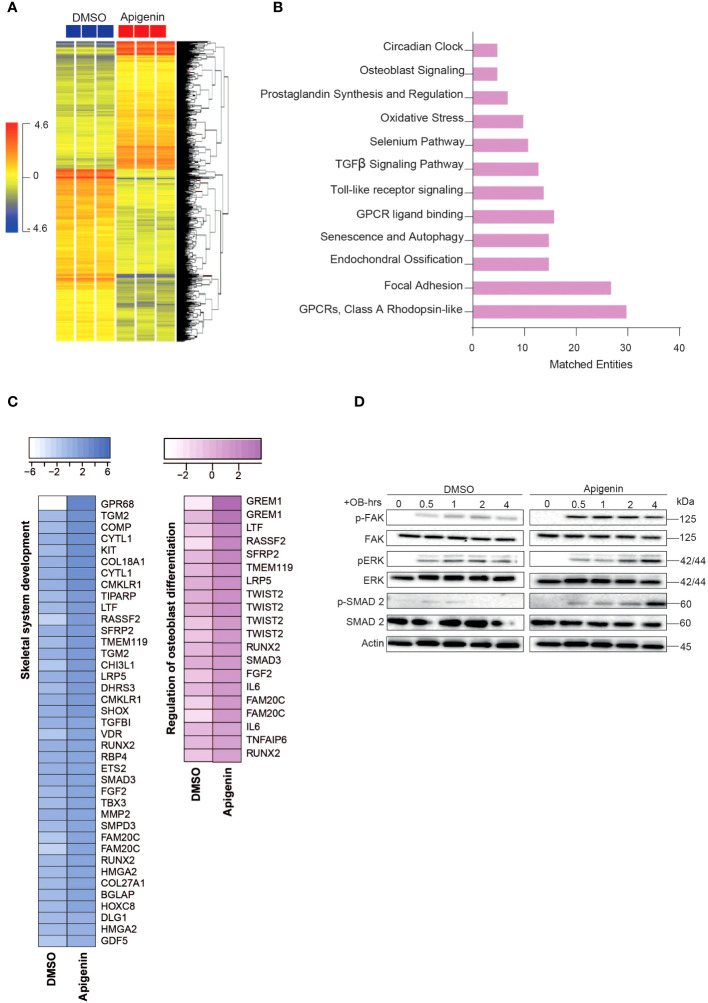
Microarray gene expression profiling of human bone marrow stromal cells (hBMSCs)-derived osteoblasts following Apigenin treatment. **(A)** Unsupervised hierarchical clustering on differentially expressed genes induced by Apigenin compared to vehicle-treated controls at day 21 following osteoblastic differentiations. **(B)** Graph illustrating the distribution of the top enriched pathways in Apigenin vs control-treated upregulated after osteoblastic differentiation of hBMSCs, where the size of the bar corresponds to the number of the matched entities. **(C)** Heat maps showing the up-regulated genes induced by Apigenin that are involved in skeletal system development, and regulation of osteoblast differentiation. **(D)** Representative western blot of hBMSCs cultures treated by Apigenin under osteogenic induction conditions on a time range from (0-4 hours), Actin was used as normal control. All results are compared to DMSO-control unless otherwise stated by the line arrow. Data without the line arrow indicates no statistical significance.

Microarray-based gene expression profiling was conducted on hBMSCs following exposure to Apigenin along with osteoblastic induction for 21 days and compared to that of vehicle-treated control cells. Hierarchical clustering based on differentially expressed transcripts showed clear separation between the Apigenin-treated and control cells (**Figure 2A**). We identified 687 upregulated and 913 downregulated transcripts (> 2.0 FC, P (corr) < 0.05; **Supplementary Table 3**). Analysis of the differentially expressed upregulated genes revealed strong enrichment for several cellular processes involved in osteoblastic differentiation, including focal adhesion, endochondral ossification, osteoblast signaling, TGFβ pathway, oxidative stress, and selenium pathway (**Figure 2B**). Heatmap for the up-regulated genes,upon Apigenin treatment, that are involved in skeletal system development and regulation of osteoblast differentiation pathways are shown (**Figure 2C**). The activation of several intracellular signaling pathways: focal adhesion kinase (FAK), extracellular signal regulated kinase (ERK) and (SMAD2) were observed upon Apigenin treatment as indicated by western blotting of p-FAK, p-ERK, and p-SMAD2 (**Figure 2D**). To identify the relevant contribution of FAK and TGFβ in Apigenin-induced osteogenesis, we tested the effects of inhibition of these pathways using FAK inhibitor (FAKi) (using PF-573228) or TGFβ inhibitor (TGFβi) (using SB505124). The results showed that Apigenin-mediated increase in ALP activity was significantly reduced by FAKi, and TGFβi (**Supplementary Figure 1A**).

#### 3.2.2 Rutaecarpine

**Figure 3:**
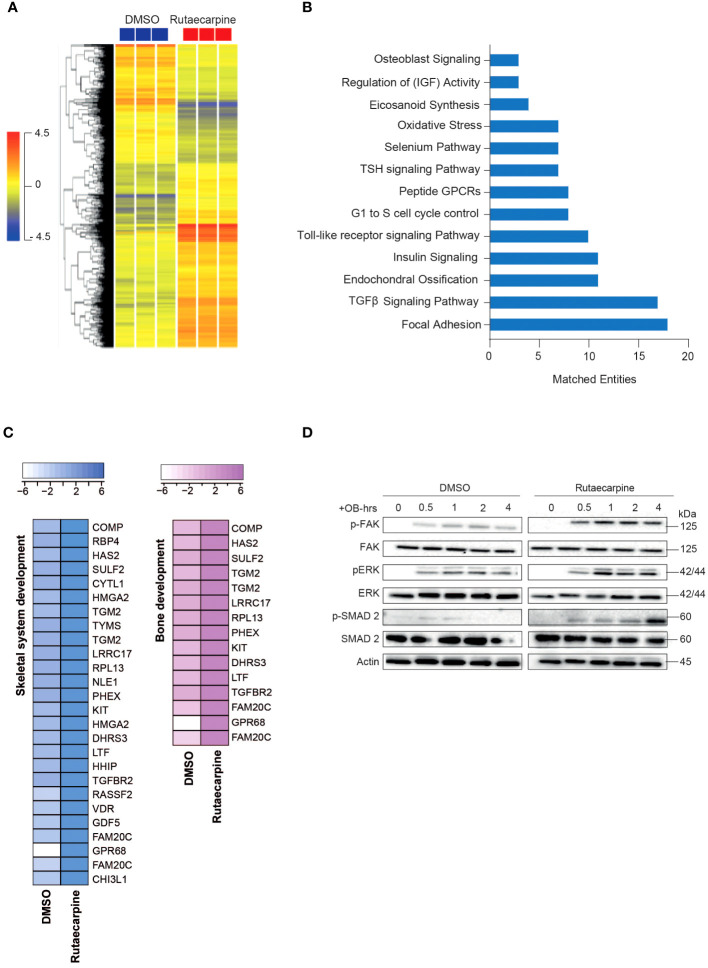
Microarray gene expression profiling of human bone marrow stromal cells (hBMSCs)-derived osteoblasts following Rutaecarpine treatment. **(A)** Unsupervised hierarchical clustering on differentially expressed genes induced by Rutaecarpine compared to vehicle-treated controls at day 21 following osteoblastic differentiations. **(B)** Graph illustrating the distribution of the top enriched pathways in Rutaecarpine vs control-treated after osteoblastic differentiation of hBMSCs where the size of the bar corresponds to the number of the matched entities. **(C)** Heat maps showing the up-regulated genes by Rutaecarpine treatment in skeletal system and bone development pathways. **(D)** Representative western blot of hBMSCs cultures treated by Rutaecarpine under osteogenic induction conditions on a time range from (0-4 hours), Actin was used as normal control. All results are compared to DMSO-control unless otherwise stated by the line arrow. Data without the line arrow indicates no statistical significance.

Microarray-based gene expression profiling was conducted on hBMSCs following exposure to Rutaecarpine along with osteoblastic induction for 21 days and compared to that of vehicle-treated control cells. Hierarchical clustering based on differentially expressed transcripts showed clear separation between the Rutaecarpine-treated and control cells (**Figure 3A**). We identified 348 upregulated and 533 downregulated transcripts (> 2.0 FC, P (corr) < 0.05; **Supplementary Table 4**). Analysis of the differentially expressed upregulated revealed strong enrichment for several cellular processes involved in osteoblastic differentiation, including focal adhesion, endochondral ossification, TGFβ pathway, Toll-like receptor pathway, oxidative stress, and selenium pathway (**Figure 3B**). Heatmap for up-regulated genes,upon Rutaecarpine treatment, that are involved in skeletal system and bone development pathways are shown (**Figure 3C**). The activation of several intracellular signaling pathways: focal adhesion kinase (FAK), extracellular signal regulated kinase (ERK) and SMAD2 was observed upon Rutaecarpine treatment as indicated by western blotting of p-FAK, p-ERK, and p-SMAD2 (**Figure 3D**). To identify the relevant contribution of FAK and TGFβ in Rutaecarpine-induced osteogenesis, we tested the effects of inhibition of these pathways using FAK inhibitor (FAKi) (using PF-573228) or TGFβ inhibitor (TGFβi) (using SB505124). The results showed that Rutaecarpine-mediated increase in ALP activity was significantly reduced by FAKi, and TGFβI (**Supplementary Figure 1B**).

### 3.3 Apigenin and Rutaecarpine reduce senescence and oxidative stress of hBMSCs

**Figure 4:**
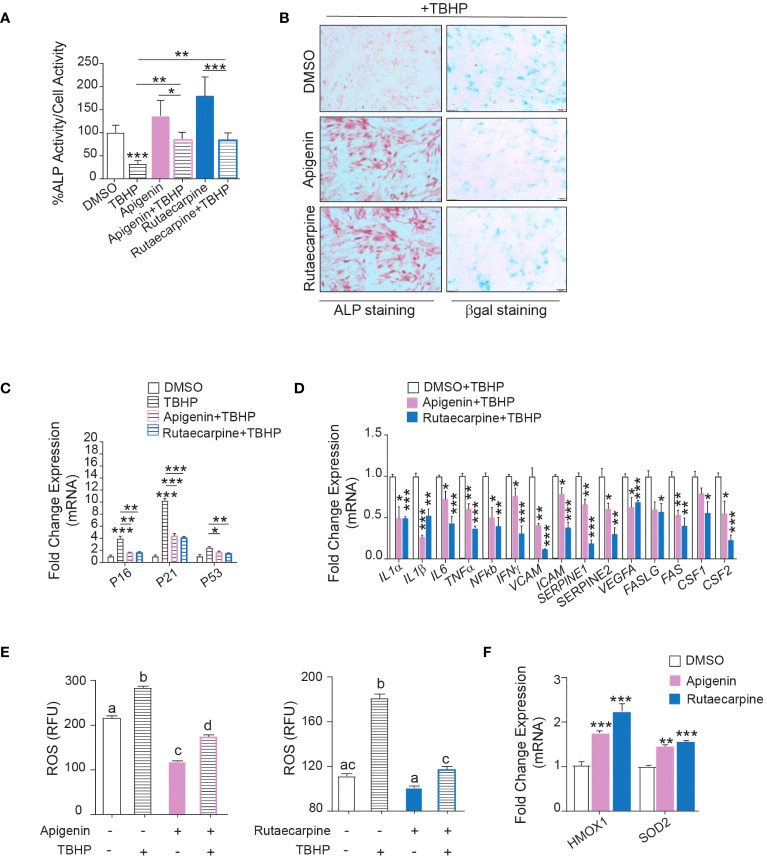
Apigenin and Rutaecarpine promote osteogenesis through downregulation of senescence and oxidative stress. Human bone marrow stromal cells (hBMSCs) were pretreated with 1µM of Apigenin or Rutaecarpine for 48 hours, followed by osteoblast differentiation. During differentiation, cells were continuously exposed to Apigenin or Rutaecarpine in the presence or absence of 50 µM of TBHP. **(A)** Quantification of ALP activity (n= 8 from two independent experiments). **(B)** ALP staining hBMSCs post treatment with vehicle control, Apigenin or Rutaecarpine, with TBHP (left panel) (4x magnification). Representative β-gal staining (right panel) in hBMSCs post treatment with vehicle control, Apigenin, or Rutaecarpine in presence of TBHP, blue cells are senescent cells (10x magnification). Gene expression was performed at day 10 and data were normalized to βactin and presented as fold change ± SEM compared with vehicle-treated controls, n=6 from 2 independent experiments. **(C)** Gene expression of senescence-associated markers (P53, P16&P21). **(D)** Gene expression of senescence associated secretory phenotype (SASP). **(E)** ROS production in Apigenin (left graph) and Rutaecarpine (right graph) as determined by DCF fluorescence. **(F)** Gene expression of antioxidant enzymes, n= 6 from 2 independent experiments. Data are presented as mean ± SEM; **(A, C, D, F)** two-tailed unpaired Student’s t test compared to control; **(E)** one-way ANOVA on which values not sharing a common letter differ significantly (*P< 0.05, **P< 0.005, ***P< 0.0005). All results are compared to DMSO-control unless otherwise stated by the line arrow. Data without the line arrow indicates no statistical significance.

Pathway analysis on the differentially expressed upregulated genes in Apigenin or Rutaecarpine vs control-treated cells revealed enrichment in the oxidative stress and Selenium pathway, suggesting a possible role for Apigenin and Rutaecarpine in regulating hBMSCs biology through their antioxidant effect (29, 30). To test this hypothesis, hBMSCs were pretreated with 1µM of Apigenin or Rutaecarpine for 48 hours, followed by osteoblast differentiation. During differentiation, cells were continuously exposed to Apigenin or Rutaecarpine in the presence or absence of 50 µM of the oxidative stress inducer, Tert-butyl hydroperoxide (TBHP) (31). ALP activity (**Figure 4A**) of hBMSCs exposed to TBHP during osteogenesis revealed negative impacts of oxidative stress on the differentiation potentials of hBMSCs that was partially rescued by the treatment with Apigenin or Rutaecarpine. Apigenin or Rutaecarpine treatment reduced senescence as visualized by β-gal staining (**Figure 4B**-right-panel) and enhanced osteogenic differentiation as shown by ALP staining (**Figure 4B**-left-panel) regardless of TBHP treatment. The mRNA levels of senescence-associated markers (P53, P21, P16) (**Figure 4C**) and senescence-associated secretory phenotype markers (SASP) (**Figure 4D**), that reflect senescence microenvironment (32), were all induced in presence of TBHP but significantly suppressed when TBHP is combined with Apigenin or Rutaecarpine. The reduced senescence in Apigenin and Rutaecarpine-treated cells was accompanied by significant reduction in the levels of reactive oxygen species (ROS) (**Figure 4E**), while the expression levels of the antioxidant enzymes (HMOX1 and SOD2) were significantly induced upon Apigenin or Rutaecarpine treatments (**Figure 4F**).

### 3.4 Apigenin and Rutaecarpine rescue osteoblast differentiation capacity in aged-primary hBMSCs

**Figure 5:**
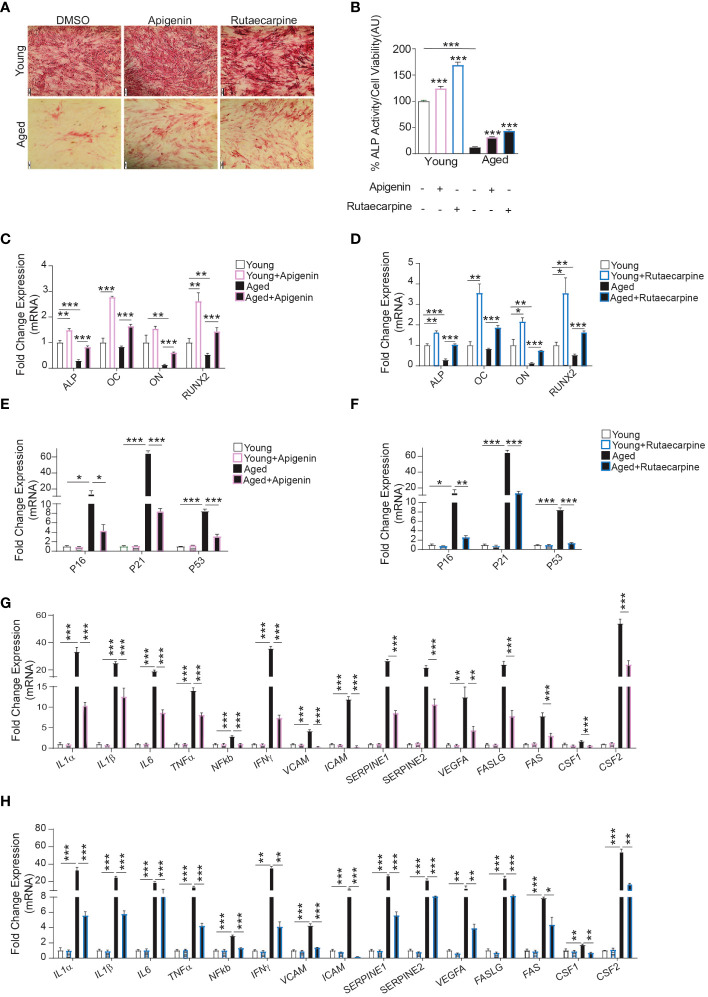
Apigenin and Rutaecarpine rescued the osteogenic differentiation phenotype of aged primary hBMSCs compared to young primary hBMSCs. Human primary bone marrow stromal cells (hBMSCs) obtained from young female donors (n=2) and aged female patients (n=2) were cultured under osteogenic differentiation supplemented with Apigenin, Rutaecarpine (1μM) or vehicle control. Cells from each participant were cultured separately, and only cells from passage two were utilized in the experiments conducted in this study. **(A)** ALP staining (4x magnification) of hBMSCs obtained from young donors (upper-panel) and old patients (lower-panel). **(B)** Quantification of ALP activity. **(C)** Gene expression of osteoblastic-specific genes post treatment with Apigenin. **(D)** Gene expression of osteoblastic-specific genes post treatment with Rutaecarpine. **(E)** Gene expression of senescence associated markers (P53, P21 &P16) post treatment with Apigenin. **(F)** Gene expression of senescence associated markers (P53, P21 &P16) post treatment with Rutaecarpine. **(G)** Gene expression of senescence-associated secretory phenotype (SASP) post treatment with Apigenin. **(H)** Gene expression of senescence-associated secretory phenotype (SASP) post treatment with Rutaecarpine. Data are presented as mean ± SEM; two-tailed unpaired Student’s t test. (*P< 0.05, **P< 0.005, ***P< 0.0005). All results are compared to young-control or aged-control unless otherwise stated by the line arrow. Data without the line arrow indicates no statistical significance. In ALP test, each subject contributed 6 technical replicates, resulting in a total of 12 observations. In qPCR, each subject contributed 4 technical replicates, resulting in a total of 8 observations.

To determine the possible therapeutic relevance of Apigenin and Rutaecarpine, we investigated the effects of Apigenin and Rutaecarpine on differentiation potentials of primary hBMSCs obtained from two young female donors and two female elderly patients. The elderly primary hBMSCs exhibited low levels of osteoblast differentiation potentials. The cells were induced to osteoblast differentiation supplementing the media with Apigenin or Rutaecarpine or vehicle control for 10 days. Apigenin or Rutaecarpine pretreatment enhanced osteoblast differentiation of the aged hBMSCs as revealed in the increase of ALP staining intensity (**Figure 5A**-lower panel) and significant increase in ALP activity (**Figure 5B**), as well as up-regulation of osteoblast differentiation marker genes in Apigenin-treated (**Figure 5C**) and Rutaecarpine-treated cells (**Figure 5D**). Interestingly, we observed significant down-regulation in gene expression of senescence-associated markers in cells treated with Apigenin (**Figure 5E**) or Rutaecarpine (**Figure 5F**) and SASP gene markers (**Figures 5G, H**). The expression levels of the antioxidant enzymes (HMOX1, SOD2 and SOD3) were induced upon Apigenin or Rutaecarpine treatments in both young and aged BMSCs-derived osteoblasts (**Supplementary Figure 2AB**).

### 3.5 Effects of Apigenin and Rutaecarpine on bone-formation in organ culture of chick femur

**Figure 6:**
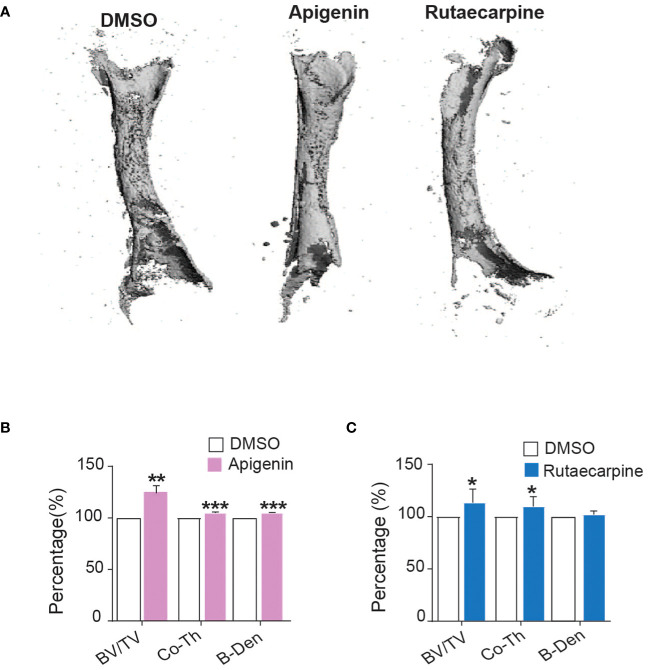
Apigenin and Rutaecarpine promoted bone-formation in chick femur model. **(A)** Representative images for μCT 3D reconstruction of bone formation in chick femur post pretreatment in Apigenin or Rutaecarpine or vehicle control in osteogenic differentiation media for 14 days, Images were analyzed at a resolution of 10.5 µm. Bone parameters were analyzed post treatment with Apigenin as bone volume per total volume (BV/TV), cortical thickness (Co.Th) and bone volume density (B.Dens). **(B)** Bone parameters were analyzed post treatment with Apigenin **(C)** Bone parameters were analyzed post treatment with Rutaecarpine. data are presented as mean fold changes ± SEM compared with vehicle-treated controls; n = 6, (*P< 0.05, **P< 0.005, ***P< 0.0005). All results are compared to DMSO-control unless otherwise stated by the line arrow. Data without the line arrow indicates no statistical significance.

Ex vivo organotypic cultures of embryonic chick femurs were used to test the impact of Apigenin and Rutaecarpine on bone formation and were scanned by μCT. Changes in bone mass were determined post 14 days of treatment of femurs with Apigenin or Rutaecarpine and compared with vehicle control DMSO (**Figure 6A**). Apigenin increased average bone volume (by ~+26% BV/TV), cortical thickness (by ~+4.8% Cort-Th) and bone density (by ~+4.8% B-Den) when compared to control (**Figure 6B**). While in Rutaecarpine, it increased the average bone volume (by ~+13.4% BV/TV), and cortical thickness (by ~+9.8% Cort-Th), but not bone density when compared to the control (**Figure 6C**).

## 4 Discussion

In the present study, we have performed a small molecule screening of a library of 143 natural compounds and identified Apigenin and Rutaecarpine for their effects on enhancing osteoblast differentiation in hBMSCs. Furthermore, we identified the possible molecular mechanisms and changes in several intracellular signaling pathways as well as their antioxidant effect against reactive oxygen species (ROS) and oxidative stress.

Several molecular pathways may explain the enhanced effects of Apigenin and Rutaecarpine on osteoblastic differentiation in hBMSCs. Apigenin and Rutaecarpine activated osteogenesis-related genes such as ALP, OC, ON and RUNX2. These genes play important roles in osteogenic maturation, matrix mineralization, and the regulation of transcription factors important for osteogenesis and bone formation (33–36). COMP is another osteogenic gene marker that was upregulated in our results with Apigenin and Rutaecarpine treatments. COMP has been shown to enhance osteogenesis via activating BMP2 and ALP activity in an ectopic bone formation rat model (37).

Microarray pathway analysis revealed enrichment and upregulation of genes involved in skeletal development, osteoblast differentiation, and bone development in cells treated with Apigenin and Rutaecarpine compared to control cells. Apigenin and Rutaecarpine activated FAK pathway which is crucial for the induction of osteogenesis and bone generation. Deficiency in this pathway has been shown to delay bone healing and interrupt mechanical stimuli in an *in vivo* tibial injury model (38, 39). Additionally, FAK inhibition blocked osterix transcriptional activity and the osteogenic differentiation of hBMSCs (40). Hu et al, reported that extracorporeal shockwave stimulation enhanced osteogenesis of hBMSCs via activation of FAK that led to activation of ERK1/2 and RUNX2. This indicates the significance of the FAK pathway in initiating the cross talk needed for osteogenesis (41). In our study, pharmacological inhibition of the FAK pathway inhibited the osteogenic induction effect of Apigenin and Rutaecarpine.

TGFβ is another upregulated pathway upon exposure of hBMSCs to Apigenin and Rutaecarpine. TGFβ regulates the postnatal bone and cartilage maintenance and recruits stromal stem cells to the bone resorption through the SMAD signaling pathway. TGFβ has been involved in coupling bone construction by osteoblasts and inducing bone destruction by osteoclastogenesis (42, 43). TGFβ isoforms and their receptors as TGFβR2 play an important signaling role in bone formation. TGFβ2 knockout mice showed lack of distal parts of the ribs (44) and transgenic mice with negative form of TGFβ2 developed hypoplastic cartilage (45). Inhibition of TGFβ pathway in our study resulted in the downregulation of the osteogenic induction effects of Apigenin and Rutaecarpine. Toll-like receptor signaling pathway was also upregulated in the presence of Apigenin and Rutaecarpine. TLRs, which are type I single-pass transmembrane proteins, have been shown to be involved in inducing osteocyte differentiation in hBMSCs. Also, activation of TLR4 promoted osteoblastic differentiation of murine MSCs through activation of WNT signaling (46).

Oxidative stress and selenium pathways are also activated in hBMSCs post treatments with Apigenin and Rutaecarpine, indicating their protective antioxidant role against age-associated bone loss. Accumulation of senescent cells and chronic up-regulation in the pro-inflammatory cytokines and SASP markers in the bone marrow microenvironment play a crucial role in age-related bone loss (47–49). Oxidative stress reduced osteogenesis of murine pre-osteoblastic (MC3T3-E1) and bone marrow-derived stromal (M2-10B4) cell lines, whereas treatments with antioxidant compounds restored the osteogenic differentiation (50). We observed that addition of exogenous H_2_O_2_ to hBMSCs downregulated osteogenesis and upregulated senescent cell accumulation, senescence-associated markers, SASP-related genes, and ROS production. These effects were all reversed by the pretreatment of cells with Apigenin and Rutaecarpine.

Primary hBMSCs from two elderly female patients exhibited low osteogenesis and higher expression of both senescence and SASP markers when compared to hBMSCs from young donors. However, treatments with Apigenin and Rutaecarpine reduced the burden of age-associated impaired osteoblast differentiation. These results indicate a potential therapeutic role of Apigenin and Rutaecarpine in reducing senescent cells and protecting against age-related bone loss.

In addition, ex vivo organotypic cultures of embryonic chick femurs with Apigenin and Rutaecarpine indicated a positive role of these compounds on bone parameters, including both BV/TV and cortical thickness. In the OVX mouse model, administration of Apigenin at 10 mg/kg at 3-day intervals for 28 days revealed a protective impact against OVX-induced trabecular bone loss, and inhibited osteoclast differentiation in mouse splenic cells (14). Rutaecarpine was also investigated for its protective role against OVX-induced bone loss in rats. Tretaments of OVX rats for 3 months with either 5 or 45 mg/kg/day of Rutaecarpine incresaed bone density, possibily due to mechansims related to osteoprotegerin induction (51).

Cardiovascular diseases are major public health problems that are positively associated with osteoporosis. Men and women with cardiovascular diseases tend to have lower bone mass density (52) and that the use of anti-osteoporotic drugs may increase the risk of cardiovascular diseases, myocardial Infarction and a stroke (53). Apigenin and Rutaecarpine were investigated earlier for their positive effect in reducing the risk of cardiac diseases, as modulators of inflammation and antioxidants (16, 54). In our investigation both compounds exhibited positive upregulation of bone formation which makes them potentially beneficial for patients with osteoporosis and cardiovascular diseases.

In summary, our findings indicate protective roles of Apigenin and Rutaecarpine in enhancing bone formation via increasing the osteoblast differeniation potential of hBMSCs and reducing the levels of oxidative stress and the burden of senescent cells. Our study suggests the need for more intervention studies to investigate the impact of small-molecule natural compounds and their potential therapeutic targeting of hBMSCs differentiation and bone formation.

## Data availability statement

The datasets presented in this study can be found in online repositories. The names of the repository/repositories and accession number(s) can be found below: https://www.ncbi.nlm.nih.gov/geo/, GSE252845.

## Ethics statement

The studies involving humans were approved by the Scientific Ethics Committee of Southern Denmark (project ID: S-20160084). The studies were conducted in accordance with the local legislation and institutional requirements. The participants provided their written informed consent to participate in this study. The animal study was approved by the Institution Review Board of King Saud University Medical College and Hospital (10-2815-IRB). The study was conducted in accordance with the local legislation and institutional requirements.

## Author contributions

DA: Writing – review & editing, Writing – original draft, Visualization, Validation, Supervision, Software, Resources, Project administration, Methodology, Investigation, Formal analysis, Data curation, Conceptualization. MO: Writing – review & editing, Writing – original draft, Visualization, Validation, Supervision, Software, Resources, Project administration, Methodology, Investigation, Formal analysis, Data curation, Conceptualization. SA: Writing – review & editing, Methodology. RV: Writing – review & editing, Methodology. ND: Writing – review & editing, Methodology. RH: Writing – review & editing, Methodology. JK: Writing – review & editing, Methodology. AS: Writing – review & editing, Methodology. AA: Writing – review & editing, Funding acquisition. NA: Writing – review & editing, Visualization, Validation, Supervision, Software, Resources, Project administration, Investigation, Funding acquisition, Formal analysis, Data curation, Conceptualization. MK: Funding acquisition, Writing – review & editing, Visualization, Validation, Supervision, Software, Resources, Project administration, Investigation, Formal analysis, Data curation, Conceptualization.

## Supplementary material

Supplementary Figure 1 Effects of Apigenin and Rutaecarpine on FAK and TGFβ signaling pathways during osteogenic differentiation of human bone marrow stromal cells hBMSCs. Quantification of ALP activity in hBMSCs pretreated with **(A)** Apigenin and **(B)** Rutaecarpine in the presence or absence of FAK inhibitor (FAKi, PF-573228, 5.0μM) or TGFβ inhibitor (TGFβi, SB505124, 5.0μM). Data are presented as mean± SEM compared with vehicle-treated controls; n = 16 from two independent experiments; (*P< 0.05, **P< 0.005, ***P< 0.0005); two-tailed unpaired Student’s t test. All results are compared to DMSO-control unless otherwise stated by the line arrow. Data without the line arrow indicates no statistical significance.

Supplementary Figure 2 Effects of Apigenin and Rutaecarpine on antioxidant enzyme gene expressions in primary hBMSCs obtained from young and aged participants. Gene expression analysis of HMOX1, SOD2, and SOD3 post treatment with **(A)** Apigenin and **(B)** Rutaecarpine. Data are presented as mean ± SEM; two-tailed unpaired Student’s t test. (*P< 0.05, **P< 0.005). All results are compared to young-control or aged-control unless otherwise stated by the line arrow. Data without the line arrow indicates no statistical significance.
