# Supplementary material for: Apigenin and Rutaecarpine reduce the burden of cellular senescence in bone marrow stromal stem cells
Source: Front Endocrinol (Lausanne). 2024 Apr 4;15:1360054. doi: 10.3389/fendo.2024.1360054 (PMC11024792; doi:10.3389/fendo.2024.1360054)
Supplement: Supplementary Figure 1 — Effects of Apigenin and Rutaecarpine on FAK and TGFβ signaling pathways during osteogenic differentiation of human bone marrow stromal cells hBMSCs. Quantification of ALP activity in hBMSCs pretreated with (A) Apigenin and (B) Rutaecarpine in the presence or absence of FAK inhibitor (FAKi, PF-573228, 5.0μM) or TGFβ inhibitor (TGFβi, SB505124, 5.0μM). Data are presented as mean± SEM compared with vehicle-treated controls; n = 16 from two independent experiments; (*P< 0.05, **P< 0.005, ***P< 0.0005); two-tailed unpaired Student’s t test. All results are compared to DMSO-control unless otherwise stated by the line arrow. Data without the line arrow indicates no statistical significance. [file DataSheet_1.pdf]

Supplementary Figure 1

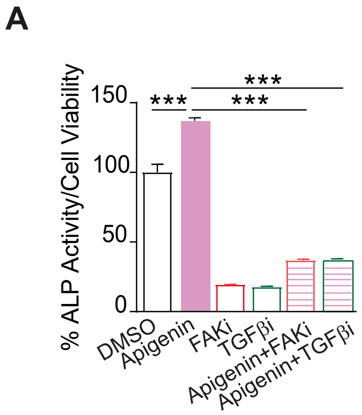

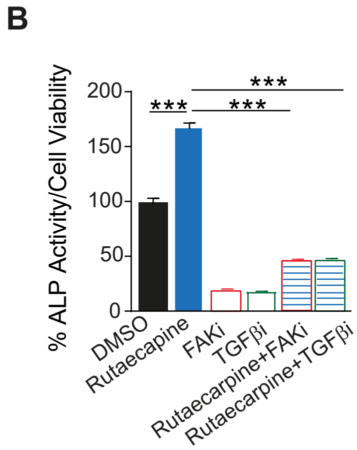

Supplementary Figure 2

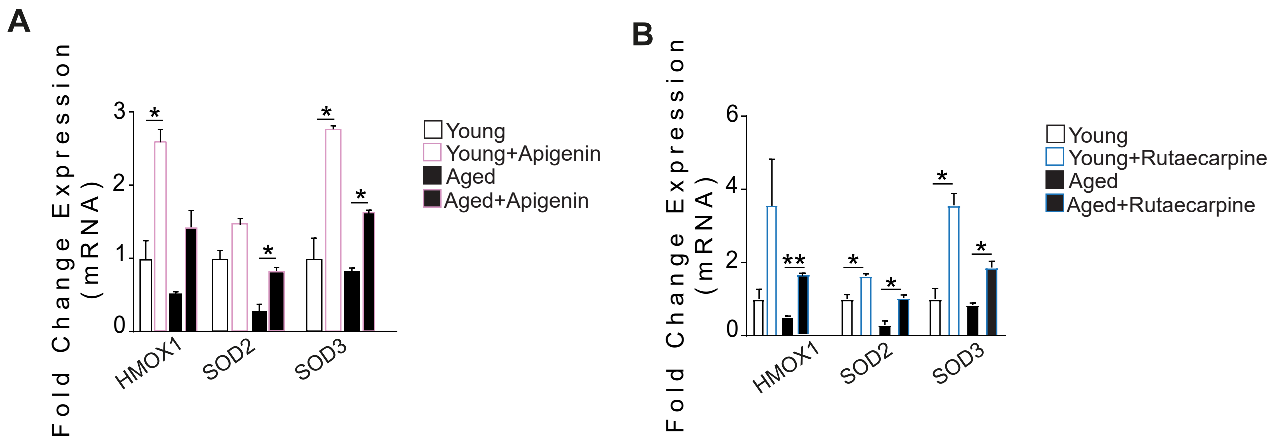

Supplementary Table 1: List of 143 natural compounds library

NO	Catalogue No	Compound Name
1	S2250	(-)-Epigallocatechin Gallate
2	S2251	(-)-Huperzine A (HupA)
3	S2252	(+)-Usniacin
4	S2253	3-Indolebutyric acid (IBA)
5	S2256	4-Methylumbelliferone (4-MU)
6	S2258	Esculin
7	S2415	Astragaloside A
8	S2260	Amygdalin
9	S2261	Andrographolide
10	S2262	Apigenin
11	S2263	Arbutin
12	S2394	Naringenin
13	S2265	Artesunate
14	S2266	Asiatic Acid
15	S2267	Azomycin
16	S2268	Baicalein
17	S2269	Baicalin
18	S2270	Bergenin
19	S2271	Berberine chloride
20	S2273	β-Sitosterol
21	S2276	Bilobalide
22	S2277	Caffeic Acid
23	S2280	Chlorogenic Acid
24	S2281	Chrysin
25	S2282	Cinchonidine
26	S2425	Apocynin
27	S2285	Cryptotanshinone
28	S2286	Cyclosporin A
29	S2287	Cytisine
30	S2422	Ipriflavone (Osteofix)
31	S2290	Dihydroartemisinin (DHA)
32	S2291	Diosgenin
33	S2292	Diosmin
34	S2293	DL-Carnitine HCl
35	S2295	Emodin
36	S2296	Enoxolone
38	S2297	Ergosterol
39	S2298	Fisetin
40	S2299	Formononetin
41	S2300	Ferulic Acid
42	S2439	Guanosine
43	S2302	Glycyrrhizin (Glycyrrhizic Acid)
44	S2303	gossypol-Acetic acid
45	S2304	Gramine
46	S2306	Gynostemma Extract
47	S2308	Hesperetin
48	S2309	Hesperidin
49	S2310	Honokiol
50	S2311	Hyodeoxycholic acid (HDCA)

Supplementary Table 1: List of 143 natural compounds library

NO	Catalogue No	Compound Name
51	S2312	Icariin
52	S2313	Indole-3-carbinol
53	S2314	Kaempferol
54	S2316	Kinetin
55	S2317	L-(+)-Rhamnose Monohydrate
56	S2319	Limonin
57	S2320	Luteolin
58	S2321	Magnolol
59	S2322	(+)-Matrine
60	S2323	Methyl-Hesperidin
61	S2324	Sodium Monensin
62	S2325	Morin Hydrate
63	S2326	Myricetin
64	S2327	Myricitrin
65	S2328	Nalidixic acid
66	S2329	Naringin
67	S3071	Vanillin
68	S2331	Neohesperidin Dihydrochalcone (Nhdc)
69	S3604	Triptolide (PG490)
70	S2333	Nobiletin
71	S2334	Oleanolic Acid
72	S2335	Oridonin
73	S2339	Paeonol
74	S2341	(-)-Parthenolide
75	S2342	Phloretin
76	S2343	Phlorizin
77	S2344	Piperine
78	S2346	Puerarin
79	S2336	Orotic acid (6-Carboxyuracil)
80	S2337	Osthole
81	S2338	Oxymatrine
82	S2347	Quercetin Dihydrate
83	S2349	Rutaecarpine
84	S2350	Rutin
85	S2351	Salicin
86	S1973	Cyclocytidine HCl
87	S2442	Inosine
88	S2423	(S)-10-Hydroxycamptothecin
89	S2354	Sclareol
90	S2355	Sclareolide
91	S2356	Shikimic Acid
92	S2358	Silymarin
93	S2359	Sinomenine
94	S2360	Solanesol (Nonaisoprenol)
95	S2362	Synephrine
96	S2363	Tangeretin
97	S2364	Tanshinone I
98	S2365	Tanshinone IIA

Supplementary Table 1: List of 143 natural compounds library

NO	Catalogue No	Compound Name
99	S2366	Taxifolin (Dihydroquercetin)
100	S2367	Tetrahydropapaverine HCl
101	S2368	Theobromine
102	S2369	Troxerutin
103	S2371	Vanillylacetone
104	S2373	Yohimbine HCl
105	S2374	5-hydroxytryptophan (5-HTP)
106	S2375	Aloin
107	S2376	Ammonium Glycyrrhizinate
108	S2377	Biochanin A
109	S2378	Butylscopolamine Bromide
110	S2383	Gastrodin
111	S1985	ATP
112	S2385	Hordenine
113	S2387	Lappaconite HBr
114	S2386	Indirubin
115	S2388	L-carnitine
116	S2389	Naringin Dihydrochalcone
117	S2390	Polydatin
118	S2391	Quercetin
119	S2392	Sesamin
120	S2393	Sorbitol
121	S2395	Rheochrysidin
122	S2396	Salidroside
123	S2397	Palmatine chloride
124	S2398	Coenzyme Q10 (CoQ10)
125	S2399	Dihydromyricetin
126	S2400	Rheic Acid
127	S2401	Sodium Danshensu
128	S2402	Cyclovirobuxin D
129	S2403	Tetrandrine
130	S2381	D-Mannitol
131	S2404	Isoliquiritigenin
132	S2405	Sophocarpine
133	S2406	Chrysophanic Acid
134	S2407	Curcumol
135	S2332	Neohesperidin
136	S2372	Xanthone
137	S2379	Dioscin
138	S2380	Diosmetin
139	S2382	Evodiamine
140	S2384	Hematoxylin
141	S2424	Hypoxanthine
142	S2437	Rotundine
143	S2357	Silibinin

Supplementary Table 2: List of primers sequences used for qRT-PCR

Primer Name	Forward sequence	Reverse sequence
Beta-actin	ATTGGCAATGAGCGGTTCCG	AGGGCAGTGATCTCCTTCTG
OC	GGCAGCGAGGTAGTGAAGAG	CTCACACACCTCCCTCCTG
ON	GAGGAAACCGAAGAGGAGG	GGGGTGTTGTTCTCATCCAG
RUNX2	TGGTTACTGTCATGGCGGGTA	TCTCAGATCGTTGAACCTTGCTA
ALPL	ACGTGGCTAAGAATGTCATC	CTGGTAGGCGATGTCCTTA
IL1α	TTAGCTTCCTGAGCAATGTG	GGTCTTCATCTTGGGCAGTC
IL1β	CTCGCCAGTGAAATGATGGCT	GTCGGAGATTCGTAGCTGGAT
IL6	GCTCCTCTGCATTGCCATTG	CATCTGGTCGGTTGTGGCT
TNFα	ATGAGCACTGAAAGCATGATCC	GAGGGCTGATTAGAGAGAGGTC
NFκb	GAACCAGGGCATACCTGTGG	TAGCCTCAGGGTACTCCATCA
IFNγ	TCGGTAACTGACTTGAATGTCCA	TCCTTTTTCGCTTCCCTGTTTT
VCAM1	CAAAGGCAGAGTACGCAAACAC	GGCTGACCAAGACGGTTGTATC
ICAM1	GCCAGGAGACACTGCAGACA	TGGCTTCGTCAGAATCACGTT
SERPINE1	GGACAGACCCTTCCTCTTTGTG	GGCCCATGAAAAGGACTGTTC
SERPINE2	GGCCAATTTCTCAGGGATGTC	TGGCTTGGTGGAACACTTCAG
VEGFA	CCCACTGAGGAGTCCAACATC	GGCCTTGGTGAGGTTTGATC
FAS	AACTTGGAAGGCCTGCATCAT	CACCTGGAGGACAGGGCTTA
FASLG	CAGTCCACCCCCTGAAAAAAA	CCTTGAGTTGGACTTGCCTGTT
CSF1	TTCAGCAAGAACTGCAACAACA	TCAGGCTTGGTCACCACATC
CSF2	TGATGGCCAGCCACTACAAG	GGGTTGCACAGGAAGTTTCC
P16	GGGTCGGGTAGAGGAGGTG	CATCATGACCTGGATCGGC
P21	GGCAGACCAGCATGACAGATT	GGCTTCCTCTTGGAGAAGATCA
P53	CCTGAGGTTGGCTCTGACTGTA	TGTTCCGTCCCAGTAGATTACCA
HMOX1	CTCAAACCTCCAAAAGCC	TCAAAAACCACCCCAACCC
SOD2	GCTCCCCGCGCTTTCTTA	GCTGGTGCCGCACACT

Supplementary Table 3: Microarray data for up- and down-regulated genes in Apigenin-treated hBMSCs

Gene Symbol	Regulation	Log FC Api_vs_CTRL
GPR68	up	10.0243435
CYP1A1	up	8.476234
SERPINB2	up	8.090169
THBD	up	7.851119
PLEKHA6	up	7.2422695
LIF	up	7.058276
SCG5	up	6.804064
SHISA2	up	6.4944086
BCL2A1	up	6.368607
HLA-DPB1	up	6.338564
GREM1	up	6.221427
CHDH	up	6.214299
IL1RN	up	6.1692457
TRPM2	up	5.8954935
NKD2	up	5.8953743
NPTX1	up	5.789449
ARL4C	up	5.6712523
GDA	up	5.6443663
IL1B	up	5.63643
CYTIP	up	5.5074983
GRIN2A	up	5.476953
CADM1	up	5.4702344
EHF	up	5.4538016
ZBED6CL	up	5.4045963
MIR155HG	up	5.322051
SLC7A5	up	5.1964827
BDKRB2	up	5.180083
VNN1	up	5.1080766
FOXQ1	up	5.103117
ADAMTS14	up	5.102833
NCKAP5	up	5.071062
NANOS1	up	5.0445127
CIITA	up	4.980095
KRT34	up	4.971934
HLA-DRA	up	4.922164
HLA-DPB1	up	4.87015
HLA-DMB	up	4.753125
RAB38	up	4.747608
OLFM2	up	4.743953
LINC00900	up	4.732439
IL19	up	4.620963
SLCO2A1	up	4.5878716
CADM1	up	4.4764233
HSPB7	up	4.4641275
BDKRB1	up	4.423379
KRTAP2-3	up	4.3543034

Gene Symbol	Regulation	Log FC Api_vs_CTRL
FCRLA	up	4.3261
ADAMTSL2	up	4.304684
ST8SIA5	up	4.295642
KCTD12	up	4.2605247
HLA-DPB1	up	4.256877
RCAN2	up	4.252878
LOC102477328	up	4.189893
MARCO	up	4.1407695
GPR110	up	4.1269894
ATP8A2	up	4.1238184
RASSF2	up	4.1188903
RARRES1	up	4.079671
KRTAP1-5	up	4.064871
NTN1	up	4.0094805
ATP1B2	up	3.973978
LOC100506688	up	3.915384
IL17RB	up	3.8916695
MYBPH	up	3.8663545
TMEM132B	up	3.8658206
HLA-DPB1	up	3.8255837
KCTD12	up	3.8237362
ENTPD3	up	3.7033973
KIT	up	3.6847467
LINC00599	up	3.6846993
TGM2	up	3.6255074
HLA-DPA1	up	3.6194115
MEOX2	up	3.6095114
KRT23	up	3.6068115
KRTAP1-5	up	3.5938535
GDA	up	3.5674963
SLC16A6	up	3.5386827
CD163	up	3.5288544
CACNA2D3	up	3.527482
TFAP2C	up	3.4984431
HLA-DRB5	up	3.4935782
BLK	up	3.405579
ELFN2	up	3.3943646
COMP	up	3.3905466
SLC37A2	up	3.377028
TYRP1	up	3.3686552
FUCA1	up	3.356166
CCL3	up	3.34783
SLC14A1	up	3.3475165
LACC1	up	3.3426623
CILP2	up	3.3221953
HLA-DRB4	up	3.3002253

Gene Symbol	Regulation	Log FC Api_vs_CTRL
CD74	up	3.2906313
KCND2	up	3.2704823
DPEP1	up	3.2515066
MPP4	up	3.2426286
APOC3	up	3.2309809
STRA6	up	3.223544
EGR2	up	3.2206864
CDH13	up	3.2072043
SAMD12	up	3.1797724
TAC1	up	3.1403832
CA4	up	3.1296926
GREM1	up	3.0831614
CHI3L1	up	3.0686193
PLCG2	up	3.0608816
GSG1	up	3.058454
PLEKHA7	up	3.0578947
LTF	up	3.0188193
TEKT5	up	3.0048614
SIM2	up	3.0025043
LINC00886	up	2.99428
FAM20C	up	2.9881818
CYP39A1	up	2.9813452
HLA-DQB1	up	2.94714
VWCE	up	2.9462705
ADORA1	up	2.9400296
FAM167A	up	2.9293568
CYP1B1	up	2.929007
IQCD	up	2.9234607
USP30-AS1	up	2.916913
AMPD3	up	2.9085937
NTM	up	2.9028635
GNAO1	up	2.9024622
NUP210P1	up	2.8956394
HLA-DRB1	up	2.8795
RPS6KA5	up	2.871821
PTPRE	up	2.8717139
PPP4R4	up	2.849889
GPR39	up	2.8498416
GPR115	up	2.8456073
EMP2	up	2.8454318
VDR	up	2.8348465
CDCP1	up	2.8301194
TMEM132B	up	2.8128388
MPP4	up	2.7788987
PECAM1	up	2.7695773
EREG	up	2.7560863

Gene Symbol	Regulation	Log FC Api_vs_CTRL
COL18A1	up	2.743011
IDO1	up	2.7389612
GPR116	up	2.7299337
TREM1	up	2.7125149
PLEKHS1	up	2.6979263
TNFSF14	up	2.69011
BEX1	up	2.6759334
C1QA	up	2.6756144
SLC7A11	up	2.6717672
LINC00673	up	2.668148
MYZAP	up	2.6667428
CYTL1	up	2.6452558
MMP7	up	2.6367311
LOXL4	up	2.6303298
CHAC1	up	2.6286607
PIP	up	2.6212351
EMP2	up	2.5589757
FAM167A	up	2.555859
COLEC12	up	2.5528357
TIPARP	up	2.5438428
SECTM1	up	2.5294974
HCG26	up	2.525295
SIPA1L2	up	2.5131903
TDRD6	up	2.5082433
RFX8	up	2.5026405
LOC100128320	up	2.5025878
GNAO1	up	2.4783597
LPXN	up	2.473866
CDCP1	up	2.4695444
CYGB	up	2.4673257
CLMN	up	2.457237
TNFRSF8	up	2.449643
ADAMTS5	up	2.449133
WFDC2	up	2.4420364
CYP1B1	up	2.434308
DAPK1	up	2.4295871
AADACL4	up	2.4295697
VEGFA	up	2.4282765
CYTL1	up	2.4208553
VEGFA	up	2.419292
TTC29	up	2.4162045
TMIGD3	up	2.4083135
NFE2	up	2.400422
IQCA1	up	2.3936555
RCAN2	up	2.3882859
FOLR3	up	2.3813775

Gene Symbol	Regulation	Log FC Api_vs_CTRL
BDH1	up	2.3684578
RFX8	up	2.3612957
TYRP1	up	2.349838
WFDC2	up	2.3452551
SGPP2	up	2.324066
HECW2	up	2.31903
TWIST2	up	2.3174458
TMEM119	up	2.309835
ALOX5AP	up	2.3053286
COL25A1	up	2.2951584
CHDH	up	2.281453
HLA-DOA	up	2.278359
LY6K	up	2.2679029
LBP	up	2.2647462
BEND5	up	2.2547104
EVI2B	up	2.2534695
KCNJ2	up	2.2488585
FRMD3	up	2.2487242
ST6GALNAC3	up	2.2482092
SHANK2	up	2.2382646
LINC01119	up	2.236314
ARHGEF3	up	2.2349286
MYO5C	up	2.222604
LINC00310	up	2.2218504
GPAM	up	2.2200289
WFDC21P	up	2.2166724
ATP8A2	up	2.2165256
TWIST2	up	2.2113342
UBD	up	2.2080824
CMKLR1	up	2.2040443
TFPI2	up	2.2025385
lnc-FAM43A-2	up	2.1985905
CLIC6	up	2.1981103
GDNF	up	2.1978314
HLA-DPA1	up	2.1907508
DEPTOR	up	2.1875343
SLA	up	2.1844878
EPHB2	up	2.180636
ALDH1A3	up	2.163042
CARD10	up	2.1559937
SFRP2	up	2.1518266
LINC00525	up	2.145011
MICAL3	up	2.1237414
SCN1A	up	2.1214738
RIN3	up	2.1173882
ZMIZ1-AS1	up	2.1085827

Gene Symbol	Regulation	Log FC Api_vs_CTRL
FAM20C	up	2.1001582
DOK7	up	2.0880692
CYTH4	up	2.0865107
F2RL3	up	2.0805829
ARNT2	up	2.079858
MAFF	up	2.0752234
DHRS3	up	2.0751598
NCCRP1	up	2.0603316
IL4I1	up	2.0571914
FAIM3	up	2.05699
CD163L1	up	2.055115
HMCN1	up	2.0529938
CDK15	up	2.049791
LBH	up	2.0497744
SLC14A1	up	2.0496755
LAMA3	up	2.0426962
SLC43A3	up	2.0415337
ARSG	up	2.0409188
GDNF	up	2.0407548
GPAM	up	2.0281353
NMNAT2	up	2.0114443
PREX1	up	2.0049918
CCL5	up	2.0046115
LINC00158	up	1.9963728
FOXF2	up	1.9936779
AQP9	up	1.9784791
AK5	up	1.9683819
GDF5	up	1.9626975
SLC2A5	up	1.9498458
SLC35F3	up	1.9384468
C9orf3	up	1.9369712
SLC22A4	up	1.9327419
NEDD4	up	1.9199401
SLC7A14	up	1.9194893
CLMN	up	1.9112165
EGR1	up	1.9053931
FGF2	up	1.9018776
IER3	up	1.8962712
PSAT1	up	1.8923488
CDA	up	1.8907883
SLC7A2	up	1.8875793
SGPP2	up	1.8859153
OSBP2	up	1.8828403
GREM2	up	1.8813887
CPNE7	up	1.8793187
SERPINE1	up	1.8779029

Gene Symbol	Regulation	Log FC Api_vs_CTRL
SLCO4C1	up	1.8729415
EVI2A	up	1.8554956
ADORA2B	up	1.8552527
FOSL1	up	1.8549783
FRMD6	up	1.8543607
LOC729040	up	1.8527548
CCR7	up	1.8501657
THBS2	up	1.8497531
AMY1C	up	1.8487121
FAM131B	up	1.84662
CYB561A3	up	1.8452191
TGM2	up	1.8435264
FGD4	up	1.841345
INHBE	up	1.8387569
GBP5	up	1.835202
STEAP3	up	1.833852
ABTB2	up	1.8327556
ARSG	up	1.8296782
CYP19A1	up	1.8275127
RGL1	up	1.8202941
RFX8	up	1.8198891
C15orf52	up	1.8137395
IQCD	up	1.8113735
TSPAN11	up	1.8100063
PLEKHS1	up	1.8042662
SLC16A14	up	1.8037721
DOCK2	up	1.8032875
DPP4	up	1.8012891
GSG1	up	1.7984366
LRIG1	up	1.7918011
LOC284561	up	1.786531
KCNJ15	up	1.7845627
ZFYVE28	up	1.7833514
SERINC2	up	1.7813106
C15orf54	up	1.7739968
SPOCK1	up	1.7732921
FRMPD4	up	1.7696491
SLC14A1	up	1.7675157
FAM43A	up	1.7672547
FRMD3	up	1.7618347
MGC20647	up	1.7615663
SLC7A14	up	1.760799
CSF3	up	1.7585231
HLA-DMA	up	1.7581156
ZG16B	up	1.7572426
TMEM171	up	1.7559808

Gene Symbol	Regulation	Log FC Api_vs_CTRL
HLA-DPB2	up	1.752749
SLC12A8	up	1.7482421
ABHD5	up	1.7465991
FGD4	up	1.7347772
SNTB1	up	1.7325151
SLC5A3	up	1.7202814
ARHGAP22	up	1.7188268
ANKRD29	up	1.7158625
STEAP3	up	1.7141399
CCL20	up	1.711535
HLA-DMA	up	1.706147
CDK5RAP2	up	1.7037513
RFX8	up	1.702904
TMEM130	up	1.7019522
JUP	up	1.7010674
SH3KBP1	up	1.6925919
RARRES2	up	1.6866351
CTSC	up	1.6846834
PADI1	up	1.6811905
CPZ	up	1.6806763
LOC100507431	up	1.6783859
IL16	up	1.6743959
EPHB2	up	1.6671708
PSD3	up	1.6569588
ARMC9	up	1.653864
PMAIP1	up	1.6538131
LAMC2	up	1.6501002
WDFY4	up	1.6483561
SYNJ2	up	1.6451509
PDE4DIP	up	1.6383983
SERPINB8	up	1.6381977
LOC100132249	up	1.6370981
TPCN1	up	1.6258006
INMT	up	1.6243075
TTC29	up	1.6235602
IER3	up	1.622438
CD7	up	1.622175
PNPLA3	up	1.6184806
AMY1C	up	1.6136607
NCR3	up	1.6081953
FGF1	up	1.6047302
NOV	up	1.5996871
GDNF	up	1.5946115
C1QTNF1-AS1	up	1.5917053
ORM1	up	1.5902613
PDIA5	up	1.5893636

Gene Symbol	Regulation	Log FC Api_vs_CTRL
MT1E	up	1.5804205
WDR66	up	1.5783244
KCNJ15	up	1.5746601
ORM2	up	1.5699229
NEK6	up	1.5688493
NOX4	up	1.5686305
NTN4	up	1.5661738
IL6	up	1.5625682
INMT	up	1.5622106
MT1L	up	1.561517
AJUBA	up	1.5606084
NALCN	up	1.5561129
ITGB2	up	1.5555866
ST6GALNAC3	up	1.5530081
MYPN	up	1.5528477
CYP19A1	up	1.5515866
PROSER2	up	1.5497833
TBX3	up	1.5468102
PLA2G4C	up	1.5450828
TWIST2	up	1.544923
HCP5	up	1.5431364
MT1B	up	1.5425421
EFTUD1	up	1.5382171
GRK5	up	1.5366902
LINC01546	up	1.5298301
IDO2	up	1.5208992
NEK6	up	1.5186925
SHOX	up	1.5156015
XDH	up	1.5130904
SLC2A1	up	1.5108157
TWIST2	up	1.5103402
TREM1	up	1.5098492
SSH1	up	1.5070745
SNORA12	up	1.5060631
ACKR3	up	1.5040164
HMBOX1	up	1.5012848
DNAH10OS	up	1.4989779
MT1HL1	up	1.4975394
ACSL5	up	1.4961655
LOC102724384	up	1.4953414
TMEM217	up	1.4948372
lnc-STEAP1B-1	up	1.4924884
GRIN2A	up	1.489283
TMEM132A	up	1.4773902
LRP5	up	1.4759749
RUNX2	up	1.4739828

Gene Symbol	Regulation	Log FC Api_vs_CTRL
C14orf132	up	1.4739168
JAKMIP2-AS1	up	1.47158
UNC5B	up	1.4698154
TGFBI	up	1.4678783
GATA6	up	1.4675347
HMGA2	up	1.4655825
THBS2	up	1.4616345
SLC7A7	up	1.460726
ARNTL2	up	1.4603834
KIF6	up	1.4573321
JUN	up	1.4522876
TMEM200A	up	1.4481374
OLFML2B	up	1.4429951
MEF2A	up	1.4413766
RPL27A	up	1.4390765
CDK5RAP2	up	1.4384835
BMF	up	1.4374617
ASNS	up	1.4321471
LOC100130938	up	1.4307747
LOC100130938	up	1.4292598
DLC1	up	1.4291785
C14orf132	up	1.4267411
DGKI	up	1.4261984
HMGA1	up	1.4260123
ITGB3	up	1.4250889
NRG1	up	1.4216937
LRRC32	up	1.4207664
GXYLT2	up	1.4196199
CA13	up	1.417749
RPL21P44	up	1.412964
CTSS	up	1.4117101
RFX8	up	1.4084282
PTGFRN	up	1.4076667
RBMY1B	up	1.40431
RBMY1B	up	1.4011598
PECAM1	up	1.3998616
LCE2A	up	1.3966775
TNFAIP6	up	1.3965665
LOC100130938	up	1.393872
LAMC2	up	1.3929577
PTGIS	up	1.3918195
SLITRK6	up	1.3914798
RBP4	up	1.3875521
OLFML2B	up	1.385962
PDIA5	up	1.3852168
ADAMTSL1	up	1.3847184

Gene Symbol	Regulation	Log FC Api_vs_CTRL
TCF7	up	1.3839895
lnc-CHIC1-2	up	1.3795531
PPP1R15A	up	1.3746393
CSF1R	up	1.3741053
ACCSL	up	1.3740783
SMPD3	up	1.372653
SH3BP4	up	1.3705825
TPCN1	up	1.3700305
RUNX2	up	1.3688416
GABRE	up	1.3628888
AGPAT9	up	1.3623464
TIAM1	up	1.3620203
THBS1	up	1.3518481
C11orf44	up	1.3510138
lnc-C21orf90-1	up	1.3509488
ETS2	up	1.3481245
LOC101927841	up	1.3469154
CPXM2	up	1.3465348
IFI30	up	1.3458381
CRABP1	up	1.3449044
ZNF664-FAM10	up	1.3439316
LINC01085	up	1.3432224
SVIL	up	1.3428435
MOK	up	1.3420879
LSM3	up	1.3408061
SH3KBP1	up	1.340141
HBEGF	up	1.3385872
BIRC3	up	1.3360543
RFX8	up	1.3357699
NRP1	up	1.3350103
TOR4A	up	1.3316908
SLC6A6	up	1.3290696
LRRC16A	up	1.3282943
ZACN	up	1.327124
SLC7A1	up	1.3181298
DSP	up	1.3163571
MYOF	up	1.3124304
SLC38A5	up	1.3113275
KCNMA1	up	1.3061337
FAM107B	up	1.305755
TLR2	up	1.3030503
SSH1	up	1.3013372
C14orf132	up	1.301311
SLC12A7	up	1.2974375
FGF1	up	1.2956011
NDST2	up	1.2934546

Gene Symbol	Regulation	Log FC Api_vs_CTRL
GNG11	up	1.2932013
RAMP1	up	1.2931159
ATP13A3	up	1.2890874
OR6C75	up	1.2871873
C8orf34	up	1.2869029
NQO1	up	1.2842183
ATF5	up	1.2837301
MOK	up	1.2818725
FRMPD4	up	1.2807039
CMKLR1	up	1.2795497
FAP	up	1.2792588
IL11	up	1.2747023
LOC101927841	up	1.2714355
CRLF2	up	1.2680223
C14orf132	up	1.2642316
MICAL2	up	1.2629526
PRKAR1B	up	1.2615203
ANK1	up	1.2593488
BGLAP	up	1.2593162
LOXL2	up	1.257937
SLC38A1	up	1.2520473
C7orf69	up	1.2509476
MLPH	up	1.2489678
CPA4	up	1.2477494
SQRDL	up	1.2458361
CASC8	up	1.2426018
RFPL4AL1	up	1.2402333
ABCC3	up	1.2392812
BICC1	up	1.2379023
APOLD1	up	1.2356409
HEG1	up	1.235434
ASPHD1	up	1.2347631
COL27A1	up	1.2337053
PIGW	up	1.2328699
lnc-UQCRFS1-9	up	1.2297797
FCN3	up	1.2286414
PKP3	up	1.2267835
MYO1D	up	1.2246838
TTC39C	up	1.224298
BOD1	up	1.2205065
CTSC	up	1.2175214
MFNG	up	1.2172115
RBPMS	up	1.2166061
MILR1	up	1.2163486
FLJ45950	up	1.2136613
PLEKHG4	up	1.2041825

Gene Symbol	Regulation	Log FC Api_vs_CTRL
LOC388242	up	1.2041713
PRICKLE1	up	1.203967
GPER1	up	1.200191
ATP8B1	up	1.1998868
ERC2	up	1.1985815
LURAP1L	up	1.1984521
STXBP5-AS1	up	1.1945767
ADAMTS6	up	1.1939976
IQCA1	up	1.1905358
TBC1D8	up	1.1844003
DBH-AS1	up	1.1815559
TNS4	up	1.1797472
MT1X	up	1.1737517
CPXM2	up	1.1714447
C15orf48	up	1.1697435
C7orf72	up	1.169024
NETO1	up	1.1662793
DLEU1	up	1.1659319
LOC101927841	up	1.165053
CFI	up	1.1646299
TRPM3	up	1.1626695
DHFR	up	1.1612968
GFPT2	up	1.1611642
SLC38A1	up	1.1609668
SLAMF8	up	1.16046
ADAMTS6	up	1.1590484
THBS1	up	1.1583997
TRIOBP	up	1.1581368
SH3BP4	up	1.1578383
CSRP2	up	1.1515305
DLEU1	up	1.1510637
HSD17B2	up	1.1490563
ZPR1	up	1.1475708
HS3ST3B1	up	1.1443452
TRIM53AP	up	1.1441785
PDZK1IP1	up	1.1428132
MYOF	up	1.1400872
FLJ43315	up	1.136455
lnc-C11orf39-3	up	1.1350806
UBIAD1	up	1.1337274
FTHL17	up	1.1319575
PMF1-BGLAP	up	1.1315205
FAM19A5	up	1.1266605
CXorf36	up	1.1235633
C10orf90	up	1.1230723
PPL	up	1.1197634

Gene Symbol	Regulation	Log FC Api_vs_CTRL
MYO1D	up	1.1185082
P2RY6	up	1.1178478
HOXA-AS2	up	1.1153593
PALMD	up	1.112553
LOC728763	up	1.1109796
IFT57	up	1.1055099
SPOCD1	up	1.1053647
CABLES1	up	1.1031194
C6orf132	up	1.1023768
SHC3	up	1.1019711
LDOC1L	up	1.100558
DSP	up	1.0995396
PAMR1	up	1.0992787
WISP2	up	1.0936586
FOLR1	up	1.0926213
CYB5A	up	1.092395
SMAD3	up	1.0919957
SLC1A5	up	1.0896702
CMAHP	up	1.0883479
CXCL1	up	1.0880485
IL6	up	1.0850109
MSX2P1	up	1.0828999
EFNA5	up	1.0779073
C14orf159	up	1.0777596
HGFAC	up	1.0748994
MMP2	up	1.0742965
MYO1B	up	1.0741525
SUSD1	up	1.0719062
LINC-PINT	up	1.0697234
CFI	up	1.0690808
MN1	up	1.0686927
ARG2	up	1.0682528
HOXC8	up	1.0669918
TMEM178B	up	1.0664659
DLG1	up	1.066451
NMB	up	1.0639733
HELB	up	1.0613575
CDC42EP2	up	1.0600413
SEMA5A	up	1.0553977
GALNT5	up	1.0551503
MAPK13	up	1.0547116
ATP13A3	up	1.0531788
CD9	up	1.0524826
LMBR1	up	1.0501528
MRPS6	up	1.0496742
SLC2A11	up	1.047342

Gene Symbol	Regulation	Log FC Api_vs_CTRL
E2F7	up	1.0456737
FJX1	up	1.0436845
STK32C	up	1.0421365
SGCD	up	1.0417558
COL6A1	up	1.0408789
PLAT	up	1.0387772
TNFRSF12A	up	1.0365348
B3GALNT1	up	1.0332564
RNF157	up	1.0293478
LOC102724384	up	1.0284982
KY	up	1.0272133
EPHA5	up	1.0267342
TPST1	up	1.0256603
NEDD4L	up	1.0247266
CYP3A7	up	1.0218172
GSTM3	up	1.0210137
ZC3H12A	up	1.0202667
KCNMA1	up	1.0199746
HMGA2	up	1.0194806
ELTD1	up	1.0186359
GRIK2	up	1.0140927
LOC100130698	up	1.0136099
LEKR1	up	1.0134015
ZC3H12C	up	1.0132333
ANK3	up	1.0105183
LBH	up	1.0087595
DMKN	up	1.0073359
ARNT2	up	1.00732
TNS3	up	1.0066228
ZNF44	up	1.0062554
MPRIP	up	1.0050918
MTHFS	up	1.0039687
VSTM4	up	1.0032306

Gene Symbol	Regulation	Log FC Api_vs_CTRL
AMT	down	-1.0009173
NRBP2	down	-1.001016
TLL1	down	-1.0011414
CFH	down	-1.0011623
F7	down	-1.0015401
SMAD9	down	-1.0033832
AP1S3	down	-1.005179
lnc-LTBP3-1	down	-1.005482
LOC101930611	down	-1.0079523
GSN	down	-1.0085053
GADD45A	down	-1.0087287
ROS1	down	-1.0089617
STOM	down	-1.0094333
SMOC1	down	-1.0094676
POLI	down	-1.0095024
ZDHHC23	down	-1.0101255
FAM110B	down	-1.0106554
DLX5	down	-1.0124946
MPPE1	down	-1.013685
FLJ37786	down	-1.0138695
SLC40A1	down	-1.0140735
AGRN	down	-1.0159755
MAP2K6	down	-1.0181032
CALCOCO2	down	-1.018786
EFCAB12	down	-1.0192757
SIX2	down	-1.019316
FCN3	down	-1.0194592
SSBP3	down	-1.0206306
HCK	down	-1.0210131
FAM160B2	down	-1.0231938
CRADD	down	-1.0256045
ACER1	down	-1.0262803
PLCE1	down	-1.0263777
TRIM35	down	-1.0268801
PAQR4	down	-1.028055
KCNMB4	down	-1.0287123
AHNAK	down	-1.0294925
LINC01573	down	-1.0306618
SESTD1	down	-1.031271
PAPSS2	down	-1.0326991
LIMCH1	down	-1.0328256
PTGDS	down	-1.0331025
FAR1	down	-1.0338637
MYL2	down	-1.0345546
ADHFE1	down	-1.0349889
LPA	down	-1.0351624

Gene Symbol	Regulation	Log FC Api_vs_CTRL
METTL7A	down	-1.0353456
lnc-MRPL14-1	down	-1.0401464
CRIP1	down	-1.043476
ARHGAP5	down	-1.0435715
ROGDI	down	-1.0450772
NRCAM	down	-1.0458468
CALML4	down	-1.0458578
PSORS1C1	down	-1.0470619
LINC00475	down	-1.0477568
NID1	down	-1.0484166
TCEAL6	down	-1.048607
TMEM190	down	-1.0487294
NR6A1	down	-1.0510372
HAR1A	down	-1.052216
ZNF396	down	-1.0537735
COX11	down	-1.0541058
PRDM1	down	-1.0544505
ARHGEF9	down	-1.0544907
ARHGAP5	down	-1.0545456
NUAK2	down	-1.0559368
ABCC6	down	-1.0577793
GDF1	down	-1.0580785
DRD4	down	-1.058865
PDLIM1	down	-1.0594014
APOL4	down	-1.0609902
MX1	down	-1.0615122
BMP4	down	-1.062456
LMOD1	down	-1.0639535
PMEL	down	-1.0658563
MAML2	down	-1.066617
STX11	down	-1.0689465
DBP	down	-1.0712216
JPH2	down	-1.0720134
INHA	down	-1.0723964
FLJ35934	down	-1.0728902
LINC00950	down	-1.0732387
SSC5D	down	-1.0742204
IFIT1	down	-1.0742472
ARHGDIB	down	-1.075119
PPP2R2B	down	-1.075649
TLE4	down	-1.0758668
WDR27	down	-1.0760059
RSAD2	down	-1.0805627
SLC27A3	down	-1.0806471
NID2	down	-1.0821182
NFU1	down	-1.0835216

Gene Symbol	Regulation	Log FC Api_vs_CTRL
FYN	down	-1.0850501
PF4V1	down	-1.085676
CBLB	down	-1.0860105
ZDHHC11	down	-1.0862747
LRRC66	down	-1.0863652
NUDT4	down	-1.0876348
AVPI1	down	-1.0882449
SUSD5	down	-1.0896534
PTGFR	down	-1.0910611
HAUS7	down	-1.09198
CAPS	down	-1.0925099
RGCC	down	-1.0926824
ZNF701	down	-1.0927503
MOCOS	down	-1.0929555
DCLK1	down	-1.0930662
CRY2	down	-1.0932953
LINC00472	down	-1.0935841
PLAC9	down	-1.0937304
LOC100131826	down	-1.0948023
SAT1	down	-1.094975
PPP1R32	down	-1.0951397
SORBS1	down	-1.09554
CP	down	-1.0972815
THEMIS2	down	-1.0974808
STK17B	down	-1.0975844
NTNG2	down	-1.0984316
FLJ22447	down	-1.0988939
SEMA3C	down	-1.0997379
KLF6	down	-1.100085
FBLN1	down	-1.1001439
FLJ46875	down	-1.1009196
CLU	down	-1.1013268
PTPLAD2	down	-1.1013523
SMAD6	down	-1.1022286
LINC01372	down	-1.1023207
ISG15	down	-1.1027516
PLCB4	down	-1.1028035
MAST1	down	-1.1034229
LOC101929715	down	-1.1050916
WFIKKN2	down	-1.1057452
CTDSPL	down	-1.107194
ID1	down	-1.1082778
CCNO	down	-1.1098714
lnc-TBC1D29-1	down	-1.1109116
HS1BP3	down	-1.1121502
TENM2	down	-1.1135607

Gene Symbol	Regulation	Log FC Api_vs_CTRL
TMSB4X	down	-1.1155467
ENDOV	down	-1.1168349
KDM4B	down	-1.1173481
ACTG2	down	-1.118058
ZBTB37	down	-1.1185622
TMEM40	down	-1.1193914
PPARGC1A	down	-1.1208409
NFRKB	down	-1.1225629
TEX19	down	-1.1228241
FMO6P	down	-1.1245087
KCNK6	down	-1.1249522
NEDD9	down	-1.1262096
FBXL14	down	-1.1262455
PALM3	down	-1.1264933
NT5M	down	-1.1266313
RNF112	down	-1.1286136
LSAMP	down	-1.1287911
DSEL	down	-1.1289855
CBX3P2	down	-1.1325028
ENC1	down	-1.1331438
FGD6	down	-1.133924
FSCN2	down	-1.1339301
FOXO6	down	-1.1343622
LINC00883	down	-1.136782
TBXAS1	down	-1.136998
ANGPT1	down	-1.139236
SIRPB1	down	-1.1407154
RELN	down	-1.141121
TDRKH	down	-1.141893
HMOX2	down	-1.1428205
lnc-SPAG1-3	down	-1.1430125
SOCS2	down	-1.1434636
PRRX1	down	-1.1440562
CEMIP	down	-1.1459352
PRR20B	down	-1.1470279
BRSK2	down	-1.1472595
RPPH1	down	-1.1473584
GPX3	down	-1.1496408
BRICD5	down	-1.150199
SCARA5	down	-1.1505038
BTBD3	down	-1.1524941
CAMKK1	down	-1.1525109
FKBP5	down	-1.1528764
MAN1C1	down	-1.153091
NDNF	down	-1.1554707
TTTY14	down	-1.1567019

Gene Symbol	Regulation	Log FC Api_vs_CTRL
P4HA3	down	-1.1571941
ANGPTL1	down	-1.1580268
SMAD9	down	-1.1589105
ANKH	down	-1.1608406
TUBB2B	down	-1.1608448
IBA57-AS1	down	-1.1618643
LINC00312	down	-1.1635139
IFITM2	down	-1.1635484
LINC01372	down	-1.1651245
LOC152286	down	-1.1662538
CDK2	down	-1.1681216
HSD17B6	down	-1.1685996
NT5E	down	-1.1689651
C19orf73	down	-1.1712165
LINC00472	down	-1.1719967
FAM76A	down	-1.1722081
ICAM5	down	-1.174424
SPTSSA	down	-1.1745429
RNF122	down	-1.1752324
CSGALNACT1	down	-1.177712
SERHL2	down	-1.1791177
HTRA3	down	-1.1802932
lnc-GOLGA8J-2	down	-1.1805673
AGBL2	down	-1.181107
HTR2A	down	-1.1815569
PPARG	down	-1.1822499
ENDOD1	down	-1.1824235
C1orf233	down	-1.1832557
STON1	down	-1.1833165
DUSP23	down	-1.1842372
KCNE4	down	-1.1854615
SLC25A35	down	-1.1855887
SLC25A36	down	-1.1856465
GPRC5B	down	-1.185663
TSPAN9	down	-1.1861601
CNTRL	down	-1.186824
STK33	down	-1.1903933
ISLR	down	-1.191278
ASIC1	down	-1.1920127
AVPI1	down	-1.1928569
IL11RA	down	-1.1939843
TRIM54	down	-1.1944684
HR	down	-1.1950889
CSGALNACT1	down	-1.1958528
SCUBE2	down	-1.1970417
BCL2L11	down	-1.1995525

Gene Symbol	Regulation	Log FC Api_vs_CTRL
RAC3	down	-1.19965
ACSS1	down	-1.199932
GRAMD3	down	-1.2007991
SLC26A6	down	-1.2034659
TSPAN14	down	-1.2039198
RCL1	down	-1.2058992
LOC728730	down	-1.206402
GPR89B	down	-1.2068602
PARD6B	down	-1.207111
ATP10A	down	-1.209366
HP	down	-1.2102624
CRP	down	-1.2114978
PDLIM7	down	-1.2118394
ENPP1	down	-1.2131896
PCDHB4	down	-1.2150526
STS	down	-1.2155914
IKZF2	down	-1.2165511
MAP4K2	down	-1.2182071
IKZF2	down	-1.2191069
PDLIM7	down	-1.2194453
IGF1	down	-1.221733
FADS2	down	-1.2221286
LOC100506753	down	-1.2231597
AQP7P3	down	-1.2242703
LINC00312	down	-1.226244
STON1	down	-1.2267884
IFITM1	down	-1.2271209
MALAT1	down	-1.2291107
RDH10	down	-1.2299317
MAMLD1	down	-1.230729
GCK	down	-1.2327914
LOC441666	down	-1.2336423
SYTL2	down	-1.2368028
SAP30	down	-1.2372754
DUSP1	down	-1.2422478
DIO2	down	-1.242331
OASL	down	-1.2434621
DENND3	down	-1.2443757
SPIRE2	down	-1.2449182
SLC4A11	down	-1.2473729
FIBIN	down	-1.2498336
LINC00176	down	-1.2509623
SPRY1	down	-1.251233
DNAJC6	down	-1.2518365
REEP4	down	-1.2528003
lnc-PERP-3	down	-1.2539618

Gene Symbol	Regulation	Log FC Api_vs_CTRL
SNCAIP	down	-1.2539746
S1PR3	down	-1.2542036
MYBPC1	down	-1.2567271
RCOR2	down	-1.2586215
RPGR	down	-1.2588559
ZFHX4-AS1	down	-1.2590711
TRIM7	down	-1.2594188
CDHR4	down	-1.2597113
CHST3	down	-1.2598989
SLC8A1	down	-1.2606117
SDC1	down	-1.2608031
CPEB2	down	-1.2619305
ELANE	down	-1.2626895
GPC6	down	-1.2639081
LOC647264	down	-1.2649871
ABCA2	down	-1.266507
GPC3	down	-1.2681979
SCAPER	down	-1.2682632
SEBOX	down	-1.2682679
PLCE1	down	-1.2684423
MCTP1	down	-1.2727755
TMEM64	down	-1.2759291
KRTCAP3	down	-1.2762525
GPR126	down	-1.2773031
OSBPL5	down	-1.2779891
USP41	down	-1.2797811
MICAL1	down	-1.280727
LOC100131048	down	-1.2824106
PARD6G	down	-1.283213
TJP2	down	-1.2833052
CNTNAP3B	down	-1.2839448
MCTP1	down	-1.2850766
LOC100289026	down	-1.2855446
PER2	down	-1.2869735
DDR1	down	-1.2874298
C1QL1	down	-1.2892818
LOC81691	down	-1.2909948
CC2D2A	down	-1.2909979
PPARG	down	-1.2929236
SLC29A1	down	-1.2945298
KAT2B	down	-1.2962639
LOC729683	down	-1.2964617
ABLIM2	down	-1.29805
ITFG3	down	-1.2984596
HAPLN3	down	-1.2987673
CCDC80	down	-1.2993348

Gene Symbol	Regulation	Log FC Api_vs_CTRL
KIF26B	down	-1.2998084
MKRN9P	down	-1.3000636
IRX3	down	-1.3010659
PTPRQ	down	-1.3014507
COL4A4	down	-1.3015752
KIAA1671	down	-1.3017654
CFH	down	-1.3019271
ABCA7	down	-1.3035064
PTGFR	down	-1.3041741
TRNP1	down	-1.3047326
IMPA2	down	-1.3076228
SAMHD1	down	-1.3094056
TTYH1	down	-1.3094122
MYO15B	down	-1.309758
ADIRF	down	-1.310011
CEACAM1	down	-1.310574
KIAA1456	down	-1.3106867
C1orf35	down	-1.3107547
BCL2L11	down	-1.3111358
GPC6	down	-1.3114184
JPH4	down	-1.3128304
MC1R	down	-1.3142228
RNF115	down	-1.3149972
FOXD2	down	-1.3156137
MICAL1	down	-1.3158097
SEPT4	down	-1.3187493
PPAP2B	down	-1.318855
TLR4	down	-1.3199357
ARRDC2	down	-1.3203664
PTGER2	down	-1.3207948
KITLG	down	-1.32167
PHF20	down	-1.322851
PLCXD1	down	-1.323861
WSB1	down	-1.3282211
LINC00922	down	-1.3291247
HPR	down	-1.3359939
ENPP1	down	-1.3362358
COL4A1	down	-1.3396424
DNAJC1	down	-1.3397539
OPN3	down	-1.3424394
TPM1	down	-1.346158
TMTC1	down	-1.3476164
CDH10	down	-1.3485909
MALAT1	down	-1.3497005
MTMR11	down	-1.3503506
FMO2	down	-1.3505855

Gene Symbol	Regulation	Log FC Api_vs_CTRL
CHRDL1	down	-1.3523313
IFNGR1	down	-1.3542393
C8orf60	down	-1.355205
GSTT2	down	-1.3566043
FAM86B3P	down	-1.361628
C9orf47	down	-1.3619819
CCDC151	down	-1.362431
CDO1	down	-1.3625414
FBLN1	down	-1.3641979
NEK3	down	-1.3646464
PLCB2	down	-1.3648244
GK5	down	-1.3656013
BMP2	down	-1.3671274
CRIP2	down	-1.3704553
TMC4	down	-1.3739594
FAM89A	down	-1.3750634
AGTR1	down	-1.3752886
MFSD2A	down	-1.3754903
PDGFRB	down	-1.3770983
YJEFN3	down	-1.378597
STARD9	down	-1.3803616
ENPP3	down	-1.3804822
MYO10	down	-1.3805664
FLVCR1-AS1	down	-1.3843031
LDHC	down	-1.3852248
LOC101928837	down	-1.3861802
PER3	down	-1.3870714
ROS1	down	-1.3882976
ANKRD20A5P	down	-1.3887635
STEAP2	down	-1.3903661
OSBPL5	down	-1.3913157
CFH	down	-1.3914361
FAM134B	down	-1.3919069
FNDC3A	down	-1.3922721
GPCPD1	down	-1.394959
ZNF3	down	-1.3952385
SDC3	down	-1.3979385
NT5E	down	-1.3980093
CP	down	-1.3982637
TCEAL2	down	-1.3982818
FBLN2	down	-1.3987119
SORT1	down	-1.3987536
KANK1	down	-1.3997068
SLC7A8	down	-1.4001164
MEIS1	down	-1.4001474
DUSP5	down	-1.4004118

Gene Symbol	Regulation	Log FC Api_vs_CTRL
DSCR8	down	-1.4008281
RASIP1	down	-1.402931
NDE1	down	-1.4039162
DICER1-AS1	down	-1.4047332
STAC2	down	-1.405125
GPX6	down	-1.4090762
JADE1	down	-1.4097372
PRR5L	down	-1.411813
JAKMIP2	down	-1.4160022
PBX4	down	-1.417459
TMTC1	down	-1.4177291
ENTPD1	down	-1.4226007
NFASC	down	-1.4245464
LOC100129399	down	-1.4263815
PPP1R3C	down	-1.4279406
CFHR3	down	-1.42925
NES	down	-1.4306887
MAMLD1	down	-1.4317594
DOCK3	down	-1.4332758
PIEZO2	down	-1.4335225
PLCXD1	down	-1.4338338
IRF7	down	-1.4367648
GAGE7	down	-1.4380997
GFRA1	down	-1.4384913
LRRIQ1	down	-1.4396272
MGARP	down	-1.4423612
IL17RE	down	-1.4430227
LAMA2	down	-1.4447299
FAM183B	down	-1.4456381
GLCCI1	down	-1.4459797
LOC339192	down	-1.4480158
AQP7	down	-1.4497981
MAP4K3	down	-1.45486
CD99	down	-1.457002
CORO6	down	-1.4576383
EPHB1	down	-1.4589376
FAHD2A	down	-1.4595399
TCF21	down	-1.4612542
CCDC121	down	-1.4614394
ROR2	down	-1.4631963
HIST3H2A	down	-1.4651204
F2RL1	down	-1.4682901
ASPN	down	-1.468864
IL15	down	-1.4734426
lnc-WDR1-1	down	-1.4754283
LINC00472	down	-1.4756743

Gene Symbol	Regulation	Log FC Api_vs_CTRL
USP53	down	-1.476768
C5	down	-1.4824672
MYL9	down	-1.4830413
SHANK2-AS3	down	-1.4843006
KCNJ8	down	-1.486038
ICAM4	down	-1.4862578
LOC101927668	down	-1.4884925
FAM84A	down	-1.48963
PGF	down	-1.4913621
FNDC1	down	-1.4913653
PF4	down	-1.4914956
PPP1R14A	down	-1.4921203
SH3TC1	down	-1.4928766
ETNK2	down	-1.4939405
KLF15	down	-1.4968247
SPTLC3	down	-1.4973043
C5AR1	down	-1.4973973
DHRS9	down	-1.497926
TET1	down	-1.5007888
ITPR1	down	-1.5016267
CDH15	down	-1.503536
IMPA2	down	-1.5073439
PRR5-ARHGAP8	down	-1.5091311
TMEM238	down	-1.5100319
KGFLP2	down	-1.510825
AZGP1	down	-1.5111986
MAPK10	down	-1.5115395
GFRA1	down	-1.5193263
NBAT1	down	-1.5212222
ADIRF-AS1	down	-1.5235927
CCDC24	down	-1.5275133
SEC61A2	down	-1.5297723
ASPN	down	-1.5302726
ARHGAP5	down	-1.5312394
SLC29A4	down	-1.5315864
P3H2	down	-1.5333312
MICALCL	down	-1.5335569
OR10G4	down	-1.5354642
HIVEP3	down	-1.5366347
KIF7	down	-1.5382638
LOC100506753	down	-1.5409751
GDPD5	down	-1.5426543
RGS11	down	-1.5433674
SPTSSA	down	-1.5434759
NEDD9	down	-1.5440693
MDGA1	down	-1.5442234

Gene Symbol	Regulation	Log FC Api_vs_CTRL
AQP7P1	down	-1.5469615
C1orf87	down	-1.5490748
LINC01279	down	-1.5508394
FUOM	down	-1.5535451
LINC01554	down	-1.5596856
HIPK2	down	-1.5604359
CHRDL1	down	-1.5625403
C9orf72	down	-1.5626657
FOXO1	down	-1.5634097
TNFSF10	down	-1.5639216
HAS1	down	-1.5641828
PTPRG	down	-1.5721848
SLCO1B3	down	-1.5747825
CPEB1	down	-1.5779841
PCDH18	down	-1.5828104
HES7	down	-1.5837984
LOC653712	down	-1.5854452
WFDC3	down	-1.585798
FAM84A	down	-1.586032
COL7A1	down	-1.5864291
EDIL3	down	-1.5870469
PITPNM1	down	-1.5902956
GALNT12	down	-1.5909808
VMO1	down	-1.5945064
SH3BP5	down	-1.5955198
FIBCD1	down	-1.5960691
TFPI	down	-1.598356
POM121L10P	down	-1.6031651
F2R	down	-1.6078438
BTG2	down	-1.6087013
AGT	down	-1.6106952
LYPD6	down	-1.6140196
FAM13A-AS1	down	-1.6167654
PMS2	down	-1.6195644
LINC00472	down	-1.6235011
LIN7A	down	-1.6243325
SYCE3	down	-1.6272088
LOC145837	down	-1.6315085
CNR1	down	-1.6319098
PEX11A	down	-1.6327128
DAB2IP	down	-1.6358333
RASD1	down	-1.6383593
CHMP1B	down	-1.6392953
LOC400558	down	-1.6394963
LIMS2	down	-1.6413579
LOC101928858	down	-1.6431365

Gene Symbol	Regulation	Log FC Api_vs_CTRL
XLOC_l2_00773	down	-1.6433231
POTEF	down	-1.6437383
MALL	down	-1.6457038
PRSS23	down	-1.6541356
NFASC	down	-1.6563158
EGFEM1P	down	-1.6563305
USP53	down	-1.6563623
PTGER3	down	-1.6593171
APOL1	down	-1.6627195
MYCBPAP	down	-1.6633251
AZU1	down	-1.6636541
PHC1	down	-1.6646336
NPAS1	down	-1.6646583
APOL2	down	-1.6660178
FBXO32	down	-1.667901
CSRP1	down	-1.6690637
TMTC1	down	-1.672287
ISG20	down	-1.6722999
MAGI2	down	-1.6738467
SCUBE3	down	-1.6787378
FCRL6	down	-1.6794665
ALDH1A1	down	-1.6836401
MCAM	down	-1.6885142
MYO15B	down	-1.6898816
LPPR3	down	-1.6916753
CSPG4	down	-1.6944609
OPRL1	down	-1.6953833
LDLRAD3	down	-1.6960516
CRP	down	-1.6976184
ALPL	down	-1.6996127
LOC399715	down	-1.7092813
CORIN	down	-1.71015
ABCC9	down	-1.710495
TMEM64	down	-1.7169992
NRCAM	down	-1.7215333
GYG2	down	-1.7284815
TLR4	down	-1.7362534
IGF2	down	-1.7368684
BMP6	down	-1.742084
RTN4RL1	down	-1.7421443
ACSS1	down	-1.7462254
PPM1J	down	-1.746949
TIMP4	down	-1.7490716
TRIB2	down	-1.7503146
C1QTNF2	down	-1.752343
SLC16A4	down	-1.7606866

Gene Symbol	Regulation	Log FC Api_vs_CTRL
OBSCN	down	-1.7614474
PDE3A	down	-1.7622246
AOC3	down	-1.7654414
PRR16	down	-1.7661853
PEX5L	down	-1.7673635
JADE1	down	-1.7681124
LOC100130417	down	-1.7704563
SESN1	down	-1.7714325
PENK	down	-1.772259
RNF144A	down	-1.7744287
SCML1	down	-1.7772429
GALNT1	down	-1.77823
ADAMTSL4	down	-1.7793963
OLFML2A	down	-1.7800323
SCRG1	down	-1.7827425
LINC00883	down	-1.788307
AMOT	down	-1.7907815
CMTM5	down	-1.792517
TRIM46	down	-1.7943542
SFRP1	down	-1.8021488
KLF6	down	-1.8084128
SLPI	down	-1.8128746
ITGB4	down	-1.8138753
LRRC8C	down	-1.817498
PYGB	down	-1.8177829
IGF2	down	-1.8221712
CCL13	down	-1.8253466
TOB1	down	-1.825877
STON1-GTF2A1	down	-1.8293916
CYMP	down	-1.8323599
AGAP11	down	-1.8333927
HSPA2	down	-1.8392625
ADORA2A	down	-1.8418604
NR2F1	down	-1.8424169
DENND3	down	-1.8440554
CHSY3	down	-1.8513123
LIFR	down	-1.8523719
TNFRSF19	down	-1.8544192
CHST6	down	-1.8547661
ATP7A	down	-1.855197
lnc-FZD4-1	down	-1.8664838
MEIS1	down	-1.8666377
LRRC73	down	-1.8679516
ANKFN1	down	-1.8686458
MBL1P	down	-1.8700014
RGAG4	down	-1.8778664

Gene Symbol	Regulation	Log FC Api_vs_CTRL
KLF2	down	-1.88642
TMEM155	down	-1.8867344
HIF3A	down	-1.8892691
C1orf167	down	-1.902117
TPRG1	down	-1.9028198
CDH6	down	-1.9029651
DUSP5P1	down	-1.9056613
PALM	down	-1.910494
LMO3	down	-1.912228
LRFN4	down	-1.9145231
MMP12	down	-1.9186614
NIPAL2	down	-1.9190098
SPP1	down	-1.9220787
GPD1L	down	-1.9263301
PFKFB4	down	-1.9310449
C9orf47	down	-1.9330788
DHRS12	down	-1.934222
FCRL6	down	-1.9351938
lnc-FZD4-1	down	-1.9379778
FAM198B	down	-1.9416053
LOC101928858	down	-1.942024
HAS1	down	-1.9449105
FBXO24	down	-1.9456894
ABCC9	down	-1.9473418
PC	down	-1.9474196
PCDH18	down	-1.9511878
STC1	down	-1.9569516
C7	down	-1.9594188
DHCR24	down	-1.9622763
TRIM7	down	-1.9627938
GALNT15	down	-1.9731348
SORBS1	down	-1.9762739
CFD	down	-1.9821992
ACTA2	down	-1.9832982
TNNI2	down	-1.9844384
MARC1	down	-1.985204
BTBD11	down	-1.9934423
OSR2	down	-1.9999747
LOC100130476	down	-2.0021896
LOC441081	down	-2.0022013
CELF2	down	-2.0025754
MATN3	down	-2.0036266
LOC644838	down	-2.0065248
GABBR2	down	-2.0094733
ATP8B4	down	-2.0120475
GALNT1	down	-2.013404

Gene Symbol	Regulation	Log FC Api_vs_CTRL
PISD	down	-2.0196142
POM121L4P	down	-2.0269206
KCNIP3	down	-2.0364292
IGSF1	down	-2.0408468
SPOCK3	down	-2.041979
B3GALT4	down	-2.042254
CKB	down	-2.0476189
HAS1	down	-2.047999
PRR36	down	-2.0480466
H19	down	-2.0494156
B3GALT2	down	-2.0501206
POM121L9P	down	-2.051733
ACADL	down	-2.052008
RASSF4	down	-2.055162
PIEZO2	down	-2.0635688
RASSF4	down	-2.0720682
CIDECP	down	-2.0731087
HR	down	-2.074821
FST	down	-2.081993
ARHGAP5-AS1	down	-2.0878615
TLE2	down	-2.1002119
NEURL1B	down	-2.10576
DNAH2	down	-2.1102118
TNFSF10	down	-2.1161485
COLGALT2	down	-2.123136
ACTBL2	down	-2.1362338
CCNYL2	down	-2.1412957
STON1-GTF2A1	down	-2.1497428
C1QL4	down	-2.150719
RASL10B	down	-2.1622822
LPL	down	-2.1648057
LRFN4	down	-2.165208
NOG	down	-2.1736212
HES4	down	-2.17376
POM121L1P	down	-2.1812313
MRO	down	-2.187357
FBN2	down	-2.1936848
NPR3	down	-2.193912
RGS2	down	-2.203679
FOS	down	-2.2166018
FGF10	down	-2.2183046
LOC644838	down	-2.2242649
SLC19A3	down	-2.2248921
SLC25A18	down	-2.2263186
PP12613	down	-2.2312942
RHOB	down	-2.2324104

Gene Symbol	Regulation	Log FC Api_vs_CTRL
JAG1	down	-2.2454963
IGFBP2	down	-2.2490144
ID4	down	-2.271605
PTPRO	down	-2.2731402
VCX2	down	-2.2775822
ATP8B5P	down	-2.2834952
LY75	down	-2.2850704
POM121L1P	down	-2.2894082
LOC101928858	down	-2.2928412
SPX	down	-2.2966774
PDE4D	down	-2.3026597
LOC441081	down	-2.3035178
LOC100128988	down	-2.312691
MGC32805	down	-2.3142998
CREB3L3	down	-2.319026
ANGPTL5	down	-2.321684
NFASC	down	-2.3235257
LOC728093	down	-2.3303976
ARHGAP5-AS1	down	-2.333174
EPS8L1	down	-2.3391626
C8orf87	down	-2.3424811
CADM3	down	-2.3428721
FSD1L	down	-2.3434162
OXTR	down	-2.3457227
MME	down	-2.3498702
MARC1	down	-2.3530862
EPHB6	down	-2.3635406
MATN3	down	-2.369378
JAK2	down	-2.3712707
NPY2R	down	-2.3787336
TMEM100	down	-2.383949
C11orf86	down	-2.388396
C11orf96	down	-2.3897984
IBSP	down	-2.392766
CHST7	down	-2.3937201
C6	down	-2.3967936
TLE6	down	-2.400235
GRIN3B	down	-2.4005864
TMEM159	down	-2.4113348
TENM2	down	-2.4139714
MATN2	down	-2.427356
AREG	down	-2.4288487
MME	down	-2.438742
ADRA2C	down	-2.4491305
APOL4	down	-2.4505377
CRLF1	down	-2.462225

Gene Symbol	Regulation	Log FC Api_vs_CTRL
PTPRQ	down	-2.4705882
PLXNA2	down	-2.4767027
CDKN1C	down	-2.496296
TOX	down	-2.4974592
AFF2	down	-2.4985464
PLCL1	down	-2.4998422
NPR3	down	-2.526595
TLE6	down	-2.5320177
MME	down	-2.5347424
AREG	down	-2.538807
LOC100507195	down	-2.5400338
ITIH3	down	-2.5440729
FGF12	down	-2.5549865
MARCH10	down	-2.5656457
PTH1R	down	-2.5726323
PPP1R14A	down	-2.5753076
MAOA	down	-2.5782478
PPP1R1C	down	-2.5836232
LEPR	down	-2.6081176
FAM162B	down	-2.6091063
PLCE1-AS1	down	-2.6130016
ARHGEF37	down	-2.644885
LOC648149	down	-2.646647
PLXNA4	down	-2.6502597
PKDCC	down	-2.6584651
S100P	down	-2.6622105
LEPR	down	-2.664023
GGT5	down	-2.6704144
PDE4D	down	-2.6830385
PTK2B	down	-2.6879628
ID3	down	-2.6915817
FAM153C	down	-2.7239618
PRL	down	-2.7441742
TRABD2A	down	-2.772026
ALCAM	down	-2.789021
PKDCC	down	-2.8079557
RERGL	down	-2.8168316
LOC644662	down	-2.846513
AMPH	down	-2.8536716
CLEC3B	down	-2.863699
PPAP2A	down	-2.8785756
LFNG	down	-2.8938813
MRO	down	-2.8947515
FGF7	down	-2.8982244
MAP2	down	-2.923608
LEPR	down	-2.4635396

Gene Symbol	Regulation	Log FC Api_vs_CTRL
APOL4	down	-2.9460588
DDIT4L	down	-2.946228
HEY1	down	-2.959796
RHOB	down	-2.9620433
DDIT4L	down	-3.0108573
TRABD2A	down	-3.0365617
MMP3	down	-3.04757
IL18R1	down	-3.0687635
MOB3B	down	-3.0706139
NR4A3	down	-3.0747013
RAB37	down	-3.0850406
POM121L10P	down	-3.1431832
DKK1	down	-3.1655846
OTOGL	down	-3.175466
MGAT4C	down	-3.1857352
ACKR1	down	-3.1889243
HOPX	down	-3.1928062
NEBL	down	-3.216957
ST6GAL1	down	-3.217747
ALDH1L1	down	-3.2229917
RIMS1	down	-3.2450724
OMD	down	-3.2550144
CPAMD8	down	-3.2812026
PDE1A	down	-3.2819138
AFF2	down	-3.2960253
PDE4D	down	-3.2973053
APLN	down	-3.302627
ISM1	down	-3.3044977
LDHD	down	-3.310079
A2M	down	-3.3253891
MBP	down	-3.3699665
SYN2	down	-3.401581
ANO3	down	-3.407183
RAI2	down	-3.4145966
BBOX1	down	-3.4179285
KCNB1	down	-3.426442
PDK4	down	-3.4530182
HAPLN1	down	-3.4621105
IL18R1	down	-3.4795742
GPR64	down	-3.4983199
CTGF	down	-3.512127
TMOD1	down	-3.5953135
SLC16A12	down	-3.5982113
PLIN4	down	-3.6561263
SYN2	down	-3.6579459
CCDC85A	down	-3.65864

Gene Symbol	Regulation	Log FC Api_vs_CTRL
IL1RL1	down	-3.6661735
EMCN	down	-3.693245
FOXI1	down	-3.693336
LOC101929268	down	-3.7071247
LINC00473	down	-3.7264853
RASGRP2	down	-3.7631843
AARD	down	-3.774866
GPC3	down	-3.8341668
CIDEC	down	-3.8379598
IL18R1	down	-3.8818398
RASGRP2	down	-3.889714
ABLIM2	down	-3.9321847
SYT12	down	-3.9532683
PLIN1	down	-3.9584055
CIDEC	down	-3.9766805
MGP	down	-4.095583
FGF7	down	-4.1658454
lnc-ENPP1-2	down	-4.193739
FZD8	down	-4.2600565
TUSC5	down	-4.4212537
C11orf87	down	-4.422287
H19	down	-4.4999666
PCSK1	down	-4.575068
SCARA5	down	-4.6570807
ABLIM2	down	-4.6699066
CCDC85A	down	-4.8187485
SPARCL1	down	-5.038559
MGP	down	-5.0775933
PROK1	down	-5.167056
RGS18	down	-5.380363
BRSK2	down	-5.591375
NEU4	down	-5.594191
RASGRP2	down	-5.7409234
FRZB	down	-5.968293
SUSD2	down	-6.0444
STMN2	down	-6.0552974
SUSD2	down	-6.5893154
NPY2R	down	-6.894023
IL1RL1	down	-8.430586

Supplementary Table 4: Microarray data for up & down-regulated genes in Rutaecarpine-treated hBMSCs

Gene Symbol	Regulation	Log FC Rut_vs_CTRL
GPR68	up	6.3460155
PLEKHA6	up	5.5911283
HLA-DPB1	up	5.178385
THBD	up	5.1016645
NKD2	up	4.34359
RARRES1	up	4.291237
HLA-DRA	up	4.201909
CIITA	up	4.1801677
SLC14A1	up	4.136223
ADAMTSL2	up	4.117103
ZBED6CL	up	4.0777535
BDKRB1	up	3.9969501
GDA	up	3.890697
EHF	up	3.853776
BDKRB2	up	3.7471275
HLA-DMB	up	3.6827078
HLA-DPB1	up	3.5908804
HLA-DRB5	up	3.5680444
TREM1	up	3.3657255
ARL4C	up	3.3410842
ENTPD3	up	3.325471
LOC10050668	up	3.3021035
HLA-DRB4	up	3.2943864
KCND2	up	3.1598694
LIF	up	3.1440735
SYT7	up	3.1062152
HNF1A	up	3.102442
SLC7A2	up	3.0801249
HLA-DPB1	up	3.0742173
PIP	up	3.0441964
AQP9	up	3.0020878
TRPM2	up	2.9765406
EEF1A2	up	2.921629
HLA-DPB1	up	2.9107218
HLA-DPA1	up	2.8923295
FOXQ1	up	2.8890998
HLA-DRB1	up	2.8660884
SERPINB2	up	2.8620465
RCAN2	up	2.8558507
SLCO4A1	up	2.7729251
STRA6	up	2.768072
VNN1	up	2.7257624
RAB38	up	2.6721835
SHISA2	up	2.6439617
RASSF2	up	2.629049
BCL2A1	up	2.5964186
GSG1	up	2.5772297
SLC14A1	up	2.5190964
PMAIP1	up	2.514179

Gene Symbol	Regulation	Log FC Rut_vs_CTRL
NUP210P1	up	2.4288347
NCKAP5	up	2.4080832
PAMR1	up	2.366432
MPP4	up	2.324858
GDF5	up	2.2942152
COMP	up	2.2901318
BLK	up	2.2879994
FABP3	up	2.2736511
HLA-DQB1	up	2.2669046
CYTIP	up	2.2639062
SLC14A1	up	2.2593272
CD74	up	2.2331796
KCTD12	up	2.1688423
FAM20C	up	2.1494892
DACH2	up	2.1381009
HMGA2	up	2.129113
MEOX2	up	2.1269505
CDCP1	up	2.1258743
IQCA1	up	2.1255438
MPP4	up	2.1165764
CLMN	up	2.1088874
TFPI2	up	2.1048045
NTM	up	2.0923228
FCRLA	up	2.0883727
SCG5	up	2.0883548
TYRP1	up	2.0820026
MIR155HG	up	2.068676
CADM1	up	2.0665429
FGD4	up	2.0655007
KCNJ2	up	2.062293
COLEC12	up	2.0610952
GPR39	up	2.0581982
VDR	up	2.055022
PTGIS	up	2.0435953
HAS2	up	2.0340848
LINC00673	up	2.0307603
NFE2	up	2.0209985
lnc-FAM43A-2	up	2.0089025
RPL27A	up	2.0066085
LOC10247732[illegible]	up	2.0062983
TGM2	up	1.9619995
FGD4	up	1.9516771
KIT	up	1.9442829
CDCP1	up	1.9382176
APOC3	up	1.917805
FJX1	up	1.9058182
AMPD3	up	1.8893698
HMGA1	up	1.8525637
MT1HL1	up	1.8277444

Gene Symbol	Regulation	Log FC Rut_vs_CTRL
TREM1	up	1.8247492
NANOS1	up	1.8217905
GATA6	up	1.8154383
TEKT5	up	1.814454
RBP4	up	1.8067619
CYP1A1	up	1.7997639
MT1E	up	1.7896507
KCTD12	up	1.78301
SAMD12	up	1.7637508
RGS4	up	1.752771
DOCK8	up	1.7372328
TNFSF14	up	1.7317299
CYTH4	up	1.7215693
ARHGAP22	up	1.7166034
VEGFA	up	1.7151904
DEPTOR	up	1.7148352
CDH13	up	1.7120504
TMSB15A	up	1.6993341
RCAN2	up	1.6982853
CYP1B1	up	1.6872609
RBMY1B	up	1.6837168
MT1X	up	1.6816411
HMGA2	up	1.6780918
LTF	up	1.668675
CLMN	up	1.6606107
MT1E	up	1.6533496
PECAM1	up	1.6512866
TMEM217	up	1.6427437
CYP1B1	up	1.6424282
C11orf44	up	1.6408751
SLC12A8	up	1.6318963
ABHD5	up	1.6310233
GDNF	up	1.6288245
ZG16B	up	1.6232136
NGEF	up	1.6160668
SERPINA3	up	1.6103625
AGPAT9	up	1.6080508
LINC01551	up	1.6042738
TDRD9	up	1.6029352
SH3D21	up	1.59611
ZNF385D	up	1.5867318
CPZ	up	1.5830482
KRT16P2	up	1.5748707
RBMY1B	up	1.5738667
KRT23	up	1.5594202
SERPINA3	up	1.5591875
MT1B	up	1.5435495
PLIN2	up	1.543145
VEGFA	up	1.5334338

Gene Symbol	Regulation	Log FC Rut_vs_CTRL
TIAM1	up	1.5333744
ZACN	up	1.5260713
KRT34	up	1.5196145
SVIL	up	1.5141209
LAMA3	up	1.5114523
FAM167A	up	1.5040411
MYO19	up	1.5029767
CA4	up	1.4901164
SLC26A4	up	1.4815071
STXBP6	up	1.4797968
CADM1	up	1.4758697
PREX1	up	1.4726176
HLA-DPA1	up	1.4715104
TCAF2	up	1.4705491
FRMD3	up	1.4689927
LOXL4	up	1.4678409
IL1B	up	1.4562666
GPER1	up	1.4531921
CSF1R	up	1.4511262
SGPP2	up	1.4471455
SERPINA3	up	1.4437603
EMP2	up	1.4412545
PLCG2	up	1.4393154
LACC1	up	1.4386085
LRIG1	up	1.436369
SLC43A3	up	1.4264051
MT1A	up	1.4244475
HILPDA	up	1.4221928
CHI3L1	up	1.4120401
HLA-DMA	up	1.409649
EMP2	up	1.4096268
NMNAT2	up	1.4091424
RFX8	up	1.4040561
RARRES2	up	1.4004995
CYGB	up	1.3990777
DCBLD2	up	1.3900161
IQCA1	up	1.3894031
SLC38A5	up	1.3863015
TNFRSF10D	up	1.3853729
TMEM171	up	1.3848652
PCAT19	up	1.3829933
TWIST2	up	1.379921
IPO4	up	1.3760345
TGM2	up	1.3744696
DHRS3	up	1.3705354
KRT18	up	1.3688357
LRRC32	up	1.367311
ALOX5AP	up	1.3611367
NETO2	up	1.3588538

Gene Symbol	Regulation	Log FC Rut_vs_CTRL
GPAM	up	1.3585591
MARCO	up	1.3576587
FHL2	up	1.3527637
HP	up	1.3514793
PPL	up	1.346775
ADORA1	up	1.3430047
SIPA1L2	up	1.3420138
BHLHE41	up	1.3410766
UHRF1	up	1.3387144
PKP3	up	1.3328996
ANKRD29	up	1.3302714
THBS2	up	1.3255883
RIN3	up	1.3238438
RPL28	up	1.3234391
COL25A1	up	1.3215647
GDNF	up	1.3175077
FAM20C	up	1.3139485
CDK5RAP2	up	1.311996
AADACL4	up	1.3072119
DLK2	up	1.3054781
IER3	up	1.305045
HPR	up	1.2968497
NOX4	up	1.2929242
HHIP	up	1.2818024
TMEM255B	up	1.2792829
CLIC6	up	1.2790349
LINC01119	up	1.2759162
TCF7	up	1.2751389
KIAA0101	up	1.2708011
PAX6	up	1.270096
HIST1H3B	up	1.2674378
STEAP3	up	1.2657745
TWIST2	up	1.26542
FBL	up	1.2646314
INSC	up	1.2618064
ADAMTS5	up	1.2611194
MT1M	up	1.2605417
IMPDH2	up	1.2560441
ASPHD1	up	1.2529541
MT1G	up	1.252798
RFX8	up	1.2526426
PRICKLE1	up	1.2513969
HLA-DMA	up	1.2492521
GDNF	up	1.2406852
EGR1	up	1.2368125
PLK2	up	1.2339063
SLC2A1	up	1.2250199
LOC200772	up	1.2244213
GPR116	up	1.2218924

Gene Symbol	Regulation	Log FC Rut_vs_CTRL
TOR4A	up	1.214978
HLA-DPB2	up	1.2136419
CYTL1	up	1.2105284
KRT19	up	1.2099516
EIF4B	up	1.2023371
PRRX2	up	1.2007778
SPC25	up	1.2006203
CARHSP1	up	1.1991181
RPS23	up	1.1948924
EFTUD1	up	1.1909696
ARHGEF3	up	1.1898167
MT1X	up	1.1896399
LINC01503	up	1.189077
NEK6	up	1.1885787
RPL3	up	1.1873723
SPOCK1	up	1.1871097
LRRC17	up	1.1849072
LOC728763	up	1.1847421
MAPK13	up	1.1843734
STEAP3	up	1.1823545
SULF2	up	1.180086
TYMS	up	1.1766251
STX1A	up	1.1754556
PHEX	up	1.1725678
RFPL4AL1	up	1.171989
SFPQ	up	1.1711612
AHRR	up	1.1675279
MCM2	up	1.1659188
GXYLT2	up	1.1643299
C7orf69	up	1.161452
SERPINB8	up	1.1605233
LINC01503	up	1.1585436
MCM5	up	1.1570241
SLA	up	1.1568847
TMEM200A	up	1.1561764
ERCC6L	up	1.1535982
GPATCH4	up	1.1512898
ACCSL	up	1.1464149
C14orf132	up	1.1451036
BLM	up	1.1402268
LSR	up	1.1395266
JUN	up	1.1359675
KRTAP1-5	up	1.1358558
HHAT	up	1.1335065
FANCA	up	1.1321564
ORM2	up	1.1276593
IL17D	up	1.1270188
UHRF1	up	1.1229024
CPNE7	up	1.1214217

Gene Symbol	Regulation	Log FC Rut_vs_CTRL
LOC285178	up	1.1159682
HNRNPA1L2	up	1.1129915
HSD17B2	up	1.1127734
HNRNPA1	up	1.1050899
CARD6	up	1.1021501
DLEU1	up	1.100914
ORM1	up	1.0997812
THBS2	up	1.0993485
BATF3	up	1.099299
PAICS	up	1.0991229
DCBLD2	up	1.0981672
lnc-PPA2-1	up	1.0969096
ORC1	up	1.095578
INF2	up	1.0955507
LBP	up	1.0923934
IGFBP6	up	1.0887275
HNRNPA1L2	up	1.0856738
RGS17	up	1.0841671
GPR56	up	1.0838722
ABCC3	up	1.0779828
PRKAR1B	up	1.0734032
HN1	up	1.0719123
DKC1	up	1.0707476
C1orf226	up	1.0703549
CFI	up	1.0664544
LOC10050753(	up	1.0620546
NRP1	up	1.0592309
NOP14	up	1.0557367
HIST1H4A	up	1.055661
FTHL17	up	1.0537146
IDO1	up	1.0502983
IER5L	up	1.0444894
RPS3	up	1.0439461
NOP16	up	1.0414939
FANCD2	up	1.0353761
GRB14	up	1.0345674
DTL	up	1.0342381
WFDC21P	up	1.0322989
AK4	up	1.0317187
MYBL2	up	1.028391
lnc-FAM105B-1	up	1.0241942
ABTB2	up	1.0240802
RPL3	up	1.0229746
ITGB2	up	1.0211792
GCAT	up	1.0210398
MEF2BNB	up	1.0178175
RMI2	up	1.0159034
CDT1	up	1.0154532

Gene Symbol	Regulation	Log FC Rut_vs_CTRL
ADAM19	up	1.0105762
DLGAP5	up	1.0098983
RPL13	up	1.0053115
TBL1XR1	up	1.0052228
SNRPD1	up	1.0008388
CFH	down	-1.0005895
MYO10	down	-1.0064843
EIF2AK2	down	-1.0078042
LINC00472	down	-1.0107529
PARP9	down	-1.0139222
KLF6	down	-1.01769
IMPA2	down	-1.0201532
SLC26A5	down	-1.02176
TMEM140	down	-1.028581
TMCC3	down	-1.0350374
AGPAT4-IT1	down	-1.0358472
TGFBR1	down	-1.0373447
FAM107A	down	-1.038454
OLFM1	down	-1.0389695
PDGFRB	down	-1.041011
ANKFY1	down	-1.0433742
LOC142937	down	-1.0460122
POM121L9P	down	-1.0481429
TNFRSF19	down	-1.0492346
CIDEC	down	-1.0500087
ENC1	down	-1.0540844
IFITM4P	down	-1.0561483
GPRC5B	down	-1.0578432
SYNE4	down	-1.0619345
ZNF441	down	-1.062847
SNORA26	down	-1.0633528
LINC00545	down	-1.0649723
HR	down	-1.066868
LOC10105995[illegible]	down	-1.0685846
BCAS3	down	-1.0695297
IFI35	down	-1.0703496
CCDC80	down	-1.0746453
PRDM1	down	-1.0782979
lnc-FZD4-1	down	-1.0783391
PLCXD1	down	-1.0791343
LINC00663	down	-1.0794921
FCRL6	down	-1.0834527
RCAN1	down	-1.0844729
BCL2L11	down	-1.0852205
GBP1	down	-1.0856792
PSG8	down	-1.0882224
LOC339192	down	-1.0887591
LOC10192956[illegible]	down	-1.0900216
CLSTN3	down	-1.0969683

Gene Symbol	Regulation	Log FC Rut_vs_CTRL
LPPR4	down	-1.1005673
ENPP1	down	-1.1031432
SPTSSA	down	-1.1090283
TMEM158	down	-1.1092019
PCDH10	down	-1.1098202
TJP2	down	-1.1109695
P2RX7	down	-1.1113048
HAS1	down	-1.1137955
HES6	down	-1.1139202
LINC01372	down	-1.1144011
ENPP1	down	-1.1167611
RGS2	down	-1.1184697
CDHR4	down	-1.1194507
GPC4	down	-1.1221244
NRCAM	down	-1.1236506
PLCXD1	down	-1.124031
NSF	down	-1.1280969
REEP2	down	-1.1293515
EDIL3	down	-1.1322987
MYO15B	down	-1.1359065
PTH1R	down	-1.1404415
LOC10105995[illegible]	down	-1.1421208
MAP2K3	down	-1.1439855
LFNG	down	-1.1442288
TLL1	down	-1.1483055
IFI44	down	-1.1490932
MMP1	down	-1.1525733
ATXN2	down	-1.1547273
KAT2B	down	-1.1565995
LEPROTL1	down	-1.1569176
LTB4R	down	-1.1585243
SPTSSA	down	-1.1588136
MEIS1	down	-1.1597981
FPR1	down	-1.1612859
PLEKHH2	down	-1.1629831
IGSF9B	down	-1.163723
GPD1L	down	-1.163939
CHSY3	down	-1.168157
ARHGAP27	down	-1.1704271
ITPRIP	down	-1.1768113
OPRL1	down	-1.1772324
PKDCC	down	-1.1781985
CADM3	down	-1.1809844
CKB	down	-1.1810718
NOG	down	-1.1823484
AVIL	down	-1.1824752
FAM107A	down	-1.1831561
TMEM100	down	-1.1860143
SND1-IT1	down	-1.187721

Gene Symbol	Regulation	Log FC Rut_vs_CTRL
MS4A7	down	-1.1913395
NPR3	down	-1.1923093
HES7	down	-1.1925192
SERHL2	down	-1.1939665
NTNG2	down	-1.1951971
AQP7P1	down	-1.1971855
CHRDL1	down	-1.2005957
OSR2	down	-1.2022904
NAV3	down	-1.2048032
DDX58	down	-1.2113723
ROS1	down	-1.214886
ALCAM	down	-1.2150052
SLC4A5	down	-1.216763
CFH	down	-1.2194946
MATN3	down	-1.2202147
LIFR	down	-1.2205286
ANGPTL1	down	-1.2211765
PRDM1	down	-1.2242527
CIDECP	down	-1.2270557
AGBL2	down	-1.2274766
LGALS9C	down	-1.2311559
NEDD9	down	-1.2339154
FLCN	down	-1.2360834
FAM183B	down	-1.2367017
FBLN2	down	-1.2372428
APOL6	down	-1.2372677
MALAT1	down	-1.2372961
RSPO3	down	-1.2385292
OBSL1	down	-1.238606
MGC32805	down	-1.2396877
CFHR3	down	-1.2400216
CSRP1	down	-1.240732
STEAP2	down	-1.2493705
HIPK2	down	-1.249701
FUOM	down	-1.2520624
NAV3	down	-1.2533569
SCARNA5	down	-1.2558466
LRRC66	down	-1.2564642
LINC00545	down	-1.2585615
FOXO1	down	-1.2617816
CHRDL1	down	-1.2650199
ID2	down	-1.2675539
MFSD2A	down	-1.2716647
FAM89A	down	-1.2737149
CELF2	down	-1.27717
PDPR	down	-1.2798053
THEMIS2	down	-1.2872132
CFH	down	-1.2874097
HAPLN3	down	-1.2910613

Gene Symbol	Regulation	Log FC Rut_vs_CTRL
LMCD1	down	-1.29176
IFITM1	down	-1.2925416
KIAA0125	down	-1.2958297
PPARGC1A	down	-1.2961932
IRF9	down	-1.2984016
HDAC5	down	-1.2991536
MARCH1	down	-1.3008391
C7	down	-1.303185
ENPP3	down	-1.3070581
LOC10105995[illegible]	down	-1.3122076
CIB4	down	-1.313704
TAS2R5	down	-1.316234
POM121L1P	down	-1.3200994
ID3	down	-1.3212234
RAB37	down	-1.3254753
FNDC1	down	-1.3257519
LPA	down	-1.3261955
PIEZO2	down	-1.3307681
LINC00925	down	-1.3314784
DBX1	down	-1.3323596
KRT86	down	-1.3328073
CLDND2	down	-1.3341614
GAS1	down	-1.3354411
STON1-GTF2A[illegible]	down	-1.3361742
PTPRO	down	-1.3365384
LOC10192885[illegible]	down	-1.337581
ARPC4-TTLL3	down	-1.3377701
IL15	down	-1.3409257
FNDC3A	down	-1.3495615
RASL10B	down	-1.3534807
PBX4	down	-1.3551897
LSAMP	down	-1.3571053
ABCC6	down	-1.3582343
FIBCD1	down	-1.361682
FAM65B	down	-1.3618073
APOL6	down	-1.3653071
HCK	down	-1.3659594
POTEF	down	-1.3659837
AMPH	down	-1.3664217
SOWAHD	down	-1.3691111
SCML1	down	-1.3717604
LIMS2	down	-1.3725288
POM121L1P	down	-1.3737128
LINC01554	down	-1.3738601
PSG8	down	-1.3742418
NAALAD2	down	-1.3754137
CORO6	down	-1.3757861
MAP2K6	down	-1.3770524
C5AR1	down	-1.3787526

Gene Symbol	Regulation	Log FC Rut_vs_CTRL
OR51V1	down	-1.3831924
SCAMP5	down	-1.3869506
SSC5D	down	-1.3876907
COL4A4	down	-1.3919591
TLE2	down	-1.3944862
RASSF4	down	-1.3991544
TIMP4	down	-1.4005904
POM121L10P	down	-1.4056485
PRRT4	down	-1.4064493
HIVEP3	down	-1.4160876
PSG2	down	-1.4197574
SYCE3	down	-1.4278213
STS	down	-1.4308467
MIR143HG	down	-1.430869
DNM1P46	down	-1.4340767
STON1-GTF2A1	down	-1.4346662
SLC45A4	down	-1.435918
SLC40A1	down	-1.4387842
PKDCC	down	-1.4395626
SLC16A4	down	-1.4408319
PP12613	down	-1.4412462
ACTG2	down	-1.4436809
MCAM	down	-1.4471698
STON1	down	-1.4511223
REC8	down	-1.4520664
COL7A1	down	-1.4581187
HELZ2	down	-1.4585885
GALNT15	down	-1.4610429
APLN	down	-1.4650141
UGT2B7	down	-1.4655751
HERC6	down	-1.4669704
ANGPTL5	down	-1.4700836
KCNIP3	down	-1.4703656
IL11	down	-1.472302
DDX60	down	-1.4725827
PDE3A	down	-1.476211
TOB1	down	-1.4796939
ASIC1	down	-1.4817894
EMCN	down	-1.4879081
LEPR	down	-1.4890852
PRR5-ARHGAP8	down	-1.4923748
ARHGEF37	down	-1.4928638
ADHFE1	down	-1.5036373
XAGE2	down	-1.5104911
MGARP	down	-1.5111328
FGF10	down	-1.5123194
GRAMD3	down	-1.5157388
PSG5	down	-1.519274
STC1	down	-1.5206523

Gene Symbol	Regulation	Log FC Rut_vs_CTRL
IGFBP2	down	-1.521612
PALM	down	-1.523107
DDIT4L	down	-1.5245827
ITIH4	down	-1.5307486
SNX29	down	-1.5319742
ENGASE	down	-1.5388868
HIPK2	down	-1.540619
PLD1	down	-1.5451473
TTTY20	down	-1.546462
EPHB6	down	-1.5487465
lnc-WDR1-1	down	-1.5547683
HOPX	down	-1.5549432
ZCCHC11	down	-1.5565172
NEURL1B	down	-1.557853
RASIP1	down	-1.5610213
PISD	down	-1.5663284
LOC441081	down	-1.5664477
HR	down	-1.57231
PDE4B	down	-1.5743086
KCNJ8	down	-1.5794313
STC2	down	-1.5813006
LOC10013366[illegible]	down	-1.5840304
CFD	down	-1.5844697
AZGP1	down	-1.5854627
AFF2	down	-1.5865679
LOC10028902[illegible]	down	-1.5869482
lnc-CNTN1-1	down	-1.5910764
C1orf167	down	-1.5921504
RASSF4	down	-1.5940464
FAM230B	down	-1.5962526
APOL4	down	-1.6018069
LOC284561	down	-1.6035349
C1QL4	down	-1.6037297
DAB2IP	down	-1.603991
FBN2	down	-1.607125
DDX58	down	-1.6104028
KGFLP2	down	-1.6130371
PTGER3	down	-1.6132964
TEKT2	down	-1.6137515
MYL9	down	-1.6180348
SLC7A8	down	-1.6253659
ISG20	down	-1.6304181
NOD2	down	-1.6305399
AQP7	down	-1.6361065
MAPK10	down	-1.6377923
TRIM14	down	-1.6382008
ANKFN1	down	-1.6389613
CHST7	down	-1.639238
MAMLD1	down	-1.6401584

Gene Symbol	Regulation	Log FC Rut_vs_CTRL
AOC3	down	-1.640841
ARHGAP5-AS1	down	-1.6461114
KLF2	down	-1.657452
NR4A2	down	-1.6574819
MATN2	down	-1.6624073
LRRC14B	down	-1.6649882
LOC728093	down	-1.6682421
IFIT3	down	-1.6736302
ZNF396	down	-1.6786835
RHOB	down	-1.6792449
JAKMIP2	down	-1.6823726
COL4A1	down	-1.6860125
SORT1	down	-1.6871095
CTGF	down	-1.6878841
MME	down	-1.6906772
FGF12	down	-1.692176
LOC647264	down	-1.6993456
PENK	down	-1.7047527
HTR2A	down	-1.7071073
MIR143HG	down	-1.7077708
IL1R2	down	-1.7078555
HSD17B7	down	-1.7083856
STAC2	down	-1.7131227
LEPR	down	-1.7144061
LOC284581	down	-1.7188872
DIO2	down	-1.7191782
CRP	down	-1.7191887
POM121L4P	down	-1.7204239
LOC10013041[illegible]	down	-1.7216927
CRP	down	-1.7230039
VMO1	down	-1.7235937
LEPR	down	-1.7256608
MAMLD1	down	-1.7282066
HERC5	down	-1.7318342
TLE6	down	-1.7379597
OAS3	down	-1.7431962
NKD1	down	-1.7436664
PSG9	down	-1.748687
KCNK6	down	-1.7557876
ARHGAP5-AS1	down	-1.7608696
ACKR1	down	-1.7612172
ERC2	down	-1.7612776
KHDRBS2	down	-1.7653329
ROR2	down	-1.7712784
C11orf87	down	-1.7772317
FBXO32	down	-1.7799034
H19	down	-1.7860866
ISM1	down	-1.7902993
CCDC116	down	-1.7970612

Gene Symbol	Regulation	Log FC Rut_vs_CTRL
XAF1	down	-1.8063102
FOS	down	-1.8086445
MIR143HG	down	-1.8112315
LY75	down	-1.8121436
MAGI2-IT1	down	-1.8345547
CREB3L3	down	-1.8411353
MME	down	-1.8428828
MIR143HG	down	-1.8435247
ZNF385B	down	-1.8470256
NIPAL2	down	-1.8527275
LAMA2	down	-1.855323
DENND3	down	-1.8561085
FGF7	down	-1.8613727
ACTBL2	down	-1.8747618
PDLIM7	down	-1.8760055
ITPR1	down	-1.8763522
CNN1	down	-1.8789368
RHOB	down	-1.878971
ACP5	down	-1.8809382
OAS1	down	-1.8812218
CEMIP	down	-1.8849902
IGFBP5	down	-1.8900323
MMP12	down	-1.8938802
SPINT2	down	-1.900876
lnc-SPAG1-3	down	-1.9069152
LOC644662	down	-1.9123458
LOC441081	down	-1.9140663
C11orf96	down	-1.9148574
MAP2	down	-1.9172992
OAS2	down	-1.9178298
LOC10050719[illegible]	down	-1.9188164
CHMP1B	down	-1.9208721
BZRAP1	down	-1.9224594
DKK1	down	-1.9233272
LINC00922	down	-1.9302781
ACOXL	down	-1.937581
MDGA1	down	-1.9441642
OAS2	down	-1.9474006
ATP8B5P	down	-1.9491435
LPPR3	down	-1.9603868
MX2	down	-1.9620303
ST6GAL1	down	-1.9640402
NPY2R	down	-1.9691617
HERC6	down	-1.9799647
CLEC3B	down	-1.9875156
LOC10012898[illegible]	down	-1.9878283
PPP1R1C	down	-2.0048687
DNAH2	down	-2.0077333
PDK4	down	-2.0086176

Gene Symbol	Regulation	Log FC Rut_vs_CTRL
AARD	down	-2.0095723
MME	down	-2.011085
LOC10012939S	down	-2.0114017
PSG1	down	-2.0129397
OASL	down	-2.0151064
TMEM155	down	-2.0265152
TMOD1	down	-2.0407684
FLJ22763	down	-2.0572743
LOC399715	down	-2.060584
CYMP	down	-2.0728388
TPRG1	down	-2.0758522
ASB2	down	-2.0829062
XAF1	down	-2.0840015
PPAP2A	down	-2.0869603
SPP1	down	-2.1096816
TSPY10	down	-2.1225457
B3GALT4	down	-2.123969
RGS18	down	-2.13645
ID4	down	-2.1387305
FAM213A	down	-2.139643
ISG15	down	-2.1450932
ZNF541	down	-2.1478462
IFIT1	down	-2.148708
USP41	down	-2.1508133
PDE4D	down	-2.1557174
TENM2	down	-2.177251
TLE6	down	-2.1806457
IGF1	down	-2.1809905
TENM2	down	-2.1904912
NFASC	down	-2.1926777
TSPY3	down	-2.2107806
NFASC	down	-2.222333
RBM24	down	-2.225744
ATP8B4	down	-2.2274246
GABBR2	down	-2.2354255
PTGDS	down	-2.2371087
MBL1P	down	-2.2378154
LOC648149	down	-2.2598815
SLC25A18	down	-2.2658231
IBSP	down	-2.2837005
LDHD	down	-2.2851887
TRABD2A	down	-2.2861166
TMEM190	down	-2.292094
HES4	down	-2.2994313
LMO3	down	-2.3002388
GPR126	down	-2.3113718
FZD8	down	-2.312175
LINC00312	down	-2.3180254
LOC10013158	down	-2.3195472

Gene Symbol	Regulation	Log FC Rut_vs_CTRL
COLGALT2	down	-2.322505
PIEZO2	down	-2.327096
IFI6	down	-2.3372986
LOC10192766{}	down	-2.3521872
ADRA2C	down	-2.373549
CEMIP	down	-2.3778737
ABLIM2	down	-2.4302235
ACTA2	down	-2.4335372
MBP	down	-2.4450388
FST	down	-2.4543705
HEY1	down	-2.46098
AZU1	down	-2.4804893
lnc-SPAG1-3	down	-2.5031366
B3GALT2	down	-2.5217218
CCL13	down	-2.531216
BBOX1	down	-2.5611367
TRABD2A	down	-2.5659642
CMPK2	down	-2.5940094
DENND3	down	-2.608859
MGAT4C	down	-2.6192737
OMD	down	-2.6249943
CDKN1C	down	-2.6338623
ITIH3	down	-2.641086
IL18R1	down	-2.6677766
RGCC	down	-2.6679354
CCDC85A	down	-2.672406
PLXNA4	down	-2.7036507
FGF7	down	-2.712364
CRLF1	down	-2.7138374
HSPA2	down	-2.7149897
GGT5	down	-2.7217007
C8orf60	down	-2.7356114
PDE1A	down	-2.7446356
NPY2R	down	-2.7500014
ABLIM2	down	-2.765442
OLFML2A	down	-2.771274
BST2	down	-2.7796507
MX1	down	-2.782551
BST2	down	-2.808826
LOC10013041{}	down	-2.8248096
HAPLN1	down	-2.8408294
FNDC1	down	-2.8602011
lnc-ENPP1-2	down	-2.9057922
AGT	down	-2.937956
MARCH10	down	-2.975121
MOB3B	down	-2.9752436
PPP1R14A	down	-2.9804018
RAI2	down	-2.9878793
PRL	down	-3.0318277

Gene Symbol	Regulation	Log FC Rut_vs_CTRL
AREG	down	-3.0662935
IRF7	down	-3.0816605
PDE4D	down	-3.0873687
OXTR	down	-3.0881975
AFF2	down	-3.0906677
NFASC	down	-3.1012385
NEBL	down	-3.1466699
ALDH1L1	down	-3.149357
OTOGL	down	-3.1706142
GPR64	down	-3.1875503
AREG	down	-3.1934645
IL18R1	down	-3.2045035
CPAMD8	down	-3.2275171
SCARA5	down	-3.2565162
CCDC85A	down	-3.2684445
RSAD2	down	-3.2853694
RIMS1	down	-3.2880273
FOXI1	down	-3.3771217
MAOA	down	-3.4322777
LINC00473	down	-3.4752085
SYN2	down	-3.4917672
RASGRP2	down	-3.538083
A2M	down	-3.542626
TUSC5	down	-3.6861973
SYN2	down	-3.6939332
PCSK1	down	-3.7795322
WFDC1	down	-3.7900963
IL1RL1	down	-3.8084505
GPC3	down	-3.8362749
IL18R1	down	-3.8466856
PLIN1	down	-3.8698292
MMP3	down	-3.903428
SYT12	down	-3.991137
BRSK2	down	-4.031818
SUSD2	down	-4.036144
NR4A3	down	-4.2126784
MGP	down	-4.2713876
RASGRP2	down	-4.33853
IFI27	down	-4.420532
SUSD2	down	-4.490308
SPARCL1	down	-4.767501
PROK1	down	-4.9799037
MGP	down	-5.362029
RASGRP2	down	-5.4294047
FRZB	down	-5.43766
NEU4	down	-5.858971
STMN2	down	-5.929384
IL1RL1	down	-6.1418257
